# Cross‐Carboxylation of Methanol and Other Alcohols With CO_2_ Into Asymmetric Alkyl Methyl Carbonates Over a CeO_2_ Catalyst

**DOI:** 10.1002/chem.71064

**Published:** 2026-05-01

**Authors:** Yuan Li, Zechen Li, Mizuho Yabushita, Yoshinao Nakagawa, Keiichi Tomishige

**Affiliations:** ^1^ Department of Applied Chemistry Graduate School of Engineering Tohoku University Sendai Miyagi Japan; ^2^ Faculty of Environmental Science and Engineering Kunming University of Science and Technology Kunming Yunnan China; ^3^ Division of Materials and Environment Graduate School of Science and Technology Gunma University Kiryu Gunma Japan; ^4^ Advanced Institute for Materials Research (WPI‐AIMR) Tohoku University Sendai Miyagi Japan

**Keywords:** asymmetric catalysis, asymmetric organic carbonate, carbon dioxide fixation, ceria, one‐pot synthesis, nonreductive CO_2_ conversion

## Abstract

One‐pot synthesis of asymmetric alkyl methyl carbonates from CO_2_, methanol (MeOH), and various alcohols was demonstrated using CeO_2_ and 2‐cyanopyridine as a heterogeneous catalyst and dehydrating agent, respectively. The molar ratio of MeOH to the other alcohol and structure of the latter alcohol governed the distribution of three organic carbonates, that is, asymmetric alkyl methyl carbonate, dimethyl carbonate (DMC), and dialkyl carbonate. For primary alcohols such as ethanol, 1‐propanol, and 1‐butanol, an equimolar ratio of MeOH to the other alcohol afforded the highest distribution of the asymmetric alkyl methyl carbonate. In contrast, the low reactivity of 2‐propanol due to steric hindrance from its bulky alkyl moiety enabled the preferential formation of isopropyl methyl carbonate (iPMC) with >80% distribution among the three carbonates of iPMC, DMC, and diisopropyl carbonate. The time‐course study indicated the involvement of two reaction routes for synthesizing asymmetric organic carbonates: (i) direct cross‐carboxylation of MeOH, the other alcohol, and CO_2_ and (ii) transesterification between DMC, which is formed via homo‐carboxylation of MeOH and CO_2_, and the other alcohol. In the case of iPMC synthesis, the indirect route (ii) became dominant because of the low reactivity of 2‐propanol.

## Graphical abstract

One‐pot synthesis of asymmetric organic carbonates from CO_2_, methanol, and other alcohols was demonstrated in the presence of CeO_2_ catalyst and 2‐cyanopyridine dehydrant. This reaction proceeded through two reaction pathways—direct route via cross‐carboxylation and indirect route via production of DMC and subsequent transesterification.

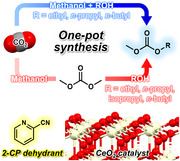

## 1 Introduction

Organic carbonates consisting of two alkyl groups are versatile and environmentally‐benign compounds that have been considered as alternatives to toxic methyl halides and phosgene as methylation and carbonylation agents [1, 2, 3, 4]. The simplest organic carbonate, dimethyl carbonate (DMC), is typically used for such objectives. Yet, asymmetric carbonates—alkyl methyl carbonates represented by ROCOOCH_3_ (R = alkyl)—are more attractive functionalization agents than DMC since a longer R group provides higher boiling point compared to DMC and offers the ease of reaction operation without pressure vessels [2, 5, 6]. In *O*‐ and *N*‐methylation, low reactivity of R group in alkyl methyl carbonates was demonstrated to be beneficial for suppressing undesired side reactions [5, 7]. For example, the chemoselectivity toward anisole in the *O*‐methylation of phenol with alkyl methyl carbonates got increased upon either or both of extension of R group and increase in bulkiness of R group because of the suppression of competitive formation of alkyl phenyl ethers (ArOR) [5]. Another intensi‐investigated application of organic carbonates is to use them as electrolytes in batteries. Symmetric carbonates such as DMC, diethyl carbonate, and ethylene carbonate have been central to this application, while asymmetric alkyl methyl carbonates such as ethyl methyl carbonate (EMC) and isopropyl methyl carbonate were demonstrated to offer both high capacity and long cycle life of graphite electrodes in Li‐ion batteries [8, 9]. These useful features of asymmetric carbonates thus make their production attractive.

The simplest asymmetric alkyl methyl carbonate is EMC. Its typical synthetic approach is transesterification between DMC and ethanol (Equation 1; R = C_2_H_5_) [10, 11, 12], the former of which can be produced from methanol (MeOH) and carbonyl sources such as phosgene [1, 13, 14], carbon monoxide [1, 14], and even CO_2_ [15, 16, 17, 18, 19].

(1)

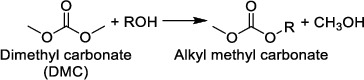




A variety of catalytic systems for this reaction at moderate reaction temperatures of 348–373 K have been reported thus far (Table S1) [20, 21, 22, 23, 24, 25, 26, 27, 28, 29, 30, 31, 32, 33]. The excess usage of DMC against ethanol is preferable for the high selectivity to EMC, and the opposite condition using excess amount of ethanol against DMC tends to decrease the EMC selectivity because of the reaction between EMC and ethanol to diethyl carbonate. The transesterification between DMC and ethanol to produce EMC was examined even in a pilot scale [12, 34]. The continuous‐flow reaction system employing a Zr‐based metal‐organic framework (MOF‐808) catalyst and reactive distillation technique was demonstrated to produce EMC in up to ∼95% yield at the column temperatures of 337 and 371 K for the top and bottom sides, respectively, where 2.5‐fold or higher equivalent amount of DMC against ethanol was employed [12]. Furthermore, the commercial‐scale production of EMC has also been announced in 2025 by UBE Corporation [35]. The same approach—transesterification between DMC and alcohol—is applicable to the synthesis of various asymmetric alkyl methyl carbonates such as methyl propyl carbonate (MPC) (Table S1) [20, 25, 36, 37, 38, 39], isopropyl methyl carbonate (iPMC) (Table S2) [25, 33, 39], and butyl methyl carbonate (BMC) (Table S1) [20, 22, 25, 33, 37, 38, 39, 40, 41, 42, 43, 44, 45]. Consistent with the synthesis of EMC (*vide supra*), excess amount of DMC against alcohol was employed for the synthesis of these asymmetric carbonates. As above, the high‐yielding synthesis of DMC is possible via the direct reaction between CO_2_ and MeOH (Equation 2) in the presence of appropriate catalyst and dehydrating agent [15, 16, 17, 18, 19], the latter of which is necessary for in situ removal of co‐produced water to shift the chemical equilibrium toward the product side.

(2)

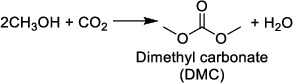




In this respect, the transesterification between DMC and alcohols is classified as an indirect and two‐pot method for using CO_2_ as a source of carbonyl moiety in asymmetric organic carbonates (Equations (1), (2)). The one‐pot conversion of CO_2_ to produce alkyl methyl carbonates has been reported by using alkyl halides (Table S3; Equation 3) [46, 47]; however, the stoichiometric amount of hydrogen halides is inevitably generated.

(3)

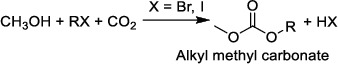




Due to the high toxicity of alkyl halides as well as the requirement of neutralization of generated hydrogen halides, a different one‐pot approach to synthesizing asymmetric organic carbonates from CO_2_ needs to be developed. Given the success that a variety of symmetric organic carbonates have been synthesized directly from CO_2_ and single alcohol [15, 16, 48, 49], reactions for a mixture of two alcohols under CO_2_ are expected to be a promising one‐pot method to synthesize asymmetric organic carbonates (Equation 4).

(4)

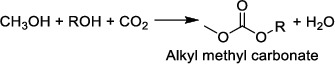




The direct synthesis of EMC from CO_2_, MeOH, and ethanol was indeed demonstrated with a CeO_2_ catalyst, yet the maximum amount of EMC was only ∼0.4 mmol at 443 K from the reaction mixture consisting of MeOH 100 mmol, ethanol 100 mmol, and CO_2_ 200 mmol [50]. Such quite low formation amount of EMC originates from the severe equilibrium limitation, which is expected to be broken by using a dehydrating agent to remove co‐produced water, consistent with the case of DMC synthesis (*vide supra*). To date, a one‐pot and high‐yielding synthesis of asymmetric organic carbonates from CO_2_ and two alcohols, including EMC, has not yet been demonstrated. For producing symmetric organic carbonates from CO_2_ and single alcohol, CeO_2_ and 2‐cyanopyridine (2‐CP) are well‐known to be an effective heterogeneous catalyst and dehydrating agent, respectively [15, 16, 48, 49, 51, 52, 53, 54, 55, 56, 57, 58, 59, 60, 61, 62]. Upon the hydration of 2‐CP, picolinamide (PA) is generated (Equation 5), which is catalyzed by CeO_2_ [63, 64, 65]. The use of 2‐CP as a dehydrating agent for nonreductive CO_2_ conversion including the production of symmetric organic carbonates has been techno‐economically assessed to be feasible by several research groups [66, 67, 68, 69, 70, 71].

(5)

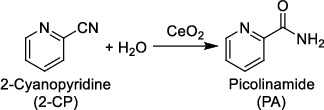




In this work, the combination of CeO_2_ and 2‐CP was, for the first time, applied to the one‐pot synthesis of alkyl methyl carbonates from CO_2_, MeOH, and various alcohols including ethanol, 1‐propanol, 2‐propanol, and 1‐butanol. Through systematic investigation of the effects of reaction parameters, we propose the reaction pathways involved in the one‐pot synthesis of asymmetric organic carbonates.

## 2 Experimental

### 2.1 Reagents

All chemicals and gases employed in this study were purchased from suppliers and used as received without further purification. Detailed information about each chemical is listed in Table S4. The catalyst employed in this study was CeO_2_ (Daiichi Kigenso Kagaku Kogyo, HS grade), which was previously reported to function as a widely applicable heterogeneous catalyst to nonreductive CO_2_ conversion [72, 73, 74, 75, 76, 77, 78, 79, 80, 81, 82, 83, 84, 85, 86, 87, 88, 89, 90, 91, 92, 93, 94, 95, 96, 97]. This catalyst was used after its calcination in air at 873 K for 3 h, and its specific surface area was 83 m^2^ g^−1^, which was determined by N_2_ physisorption measurement at 77 K (Gemini VII 2360, Micromeritics) and Brunauer‐Emmett‐Teller (BET) method.

### 2.2 Synthesis of Asymmetric Organic Carbonates From MeOH, Other Alcohols, and CO_2_

All reactions in this study to produce asymmetric organic carbonates from methanol (MeOH), other alcohols (ROH, R = alkyl group except for methyl group), and CO_2_ were carried out in a stainless‐steel autoclave reactor (HIRO Company, inner volume 190 mL) with an agitator. Typically, as substrates, methanol and ROH (total amount of 50 mmol, molar ratio of methanol to ROH = 12.5:37.5, 25:25, and 37.5:12.5) were charged into the reactor, followed by the addition of 2.0 mmol (0.34 g) of CeO_2_ catalyst, 100 mmol of acetonitrile as a solvent, 50 mmol of 2‐cyanopyridine (2‐CP) as a dehydrating agent, and a stirring bar. As ROH, ethanol (EtOH), 1‐propanol (1‐PrOH), 2‐propanol (2‐PrOH), or 1‐butanol (1‐BuOH) was used in this study. It should be noted that acetonitrile is able to behave as a dehydrating agent in the synthesis of organic carbonates from CO_2_ and alcohols to form acetamide [73], but such compound was not detected in this study due to the higher dehydrating ability of 2‐CP than that of acetonitrile. The reactor was initially purged with CO_2_ (1 MPa at room temperature (r.t.)) three times and then pressurized to 5 MPa with CO_2_ at the same temperature. The reactor was heated from r.t. to 393 K. The time when the inner temperature, which was monitored with a thermocouple inserted into the reactor, reached 393 K was defined as 0 h of reaction time in this study. The reactions were conducted for designated durations. Afterwards, the reactor was cooled down to r.t. by being immersed in a water bath. To collect the reaction mixture from the reactor completely, a certain amount of either ethanol or acetonitrile was used as a collecting solvent, followed by the addition of either 1‐hexanol or 1,4‐dioxane as an internal standard. The thus‐prepared mixture was analyzed by a gas chromatograph (GC; GC‐2014, Shimadzu) equipped with a flame ionization detector (FID) and either a CP‐Sil5 capillary column (Agilent Technology, ø0.25 mm × 50 m, film thickness 0.25 µm) or a TC‐WAX capillary column (GL Sciences, ø0.25 mm × 30 m, film thickness 0.25 µm). The conversion of alcohols, distribution of organic carbonates, selectivity to picolinamide (PA) from 2‐CP, and balance of alcohols and 2‐CP were calculated using Equations (6), (7), (8), (9), (10), (11).

(6) $$\mathrm{Conversion}\;\mathrm{of}\;\mathrm{alcohol}\;(\%)=\frac{\mathrm{Consumed}\;\mathrm{alcohol}\;(\mathrm{mol})}{\mathrm{Charged}\;\mathrm{alcohol}\;(\mathrm{mol})}\times 100$$

(7) $$\begin{array}{c} \mathrm{Distribution}\;\mathrm{of}\;\mathrm{organic}\;\mathrm{carbonate}\;(\%)\\ \;=\frac{\mathrm{Organic}\;\mathrm{carbonate}\;(\mathrm{mol})}{\sum \mathrm{Organic}\;\mathrm{carbonate}\;(\mathrm{mol})}\times 100 \end{array}$$

(8) $$\mathrm{Balance}\;\mathrm{of}\;\mathrm{alcohol}\;\left(\%\right)=\frac{\mathrm{Unreacted}\;\mathrm{alcohol}\;(\mathrm{mol})+\sum \mathrm{Corresponding}\;\mathrm{alcohol}-\mathrm{derived}\;\mathrm{moiety}\;\mathrm{in}\;\mathrm{product}\;(\mathrm{mol})}{\mathrm{Charged}\;\mathrm{alcohol}\;(\mathrm{mol})}\times 100$$

(9) $$\mathrm{Conversion}\;\mathrm{of}\;2-\mathrm{CP}\;\left(\%\right)=\frac{\mathrm{Consumed}\;2-\mathrm{CP}\;(\mathrm{mol})}{\mathrm{Charged}\;2-\mathrm{CP}\;(\mathrm{mol})}\times 100$$

(10) $$\mathrm{Selectivity}\;\mathrm{to}\;\mathrm{PA}\;\left(\%\right)=\frac{\mathrm{PA}\;(\mathrm{mol})}{\mathrm{Charged}\;2-\mathrm{CP}\left(\mathrm{mol}\right)}\times 100$$

(11) $$\mathrm{Balance}\;\mathrm{of}2-\mathrm{CP}\;\left(\%\right)=\frac{\mathrm{Unreacted}\;2-\mathrm{CP}\;(\mathrm{mol})+\sum 2-\mathrm{CP}-\mathrm{derived}\;\mathrm{moiety}\;\mathrm{in}\;\mathrm{product}\;(\mathrm{mol})}{\mathrm{Charged}\;2-\mathrm{CP}\;(\mathrm{mol})}\times 100$$

The commercially unavailable alkyl methyl carbonates—methyl propyl carbonate (MPC) and butyl metcarbonate (BMC)—were identified with a gas chromatograph equipped with a mass spectrometer (GC‐MS; GCMS‐QP2020NXC, Shimadzu, methane chemical ionization (CI)) using the same columns as above. The GC‐MS data and assignments for these compounds are shown in Figures S1 and S2. Alkyl picolinates that are commercially unavailable (i.e., ethyl picolinate, propyl picolinate, isopropyl picolinate, and butyl picolinate) were identified previously [93]. All these self‐identified compounds were quantified by GC‐FID with the consideration of their effective carbon number and building blocks derived from alcohols and 2‐CP.

To examine the reusability of CeO_2_ catalyst, the spent CeO_2_ catalyst was washed with ethanol, dried in an oven at 333 K overnight, and calcined in air at 873 K for 3 h. The resulting CeO_2_ catalyst was employed for the next reaction cycle. The spent CeO_2_ catalyst underwent thermogravimetry‐differential thermal analysis (TG‐DTA; Rigaku, Thermo Plus EVOII). In this analysis, *ca*. 10 mg of the sample was charged into a Pt pan and heat‐treated under an air flow (30 mL min^−1^) at a ramp rate of 10 K min^−1^. The calcined CeO_2_ samples were characterized by N_2_ physisorption measurement (*vide supra*) and powder X‐ray diffraction measurement (XRD; Rigaku, MiniFlex600, Cu *K*α radiation, 40 kV, 40 mA).

## 3 Results and Discussion

### 3.1 Synthesis of Ethyl Methyl Carbonate (EMC)

The initial target product in this study is ethyl methyl carbonate (EMC), which is the simplest asymmetric organic carbonate, from methanol (MeOH), ethanol (EtOH), and CO_2_ (Equation 12).

(12)

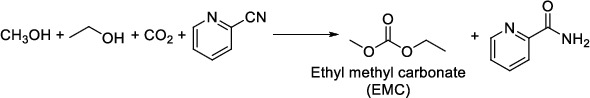




**FIGURE 1:**
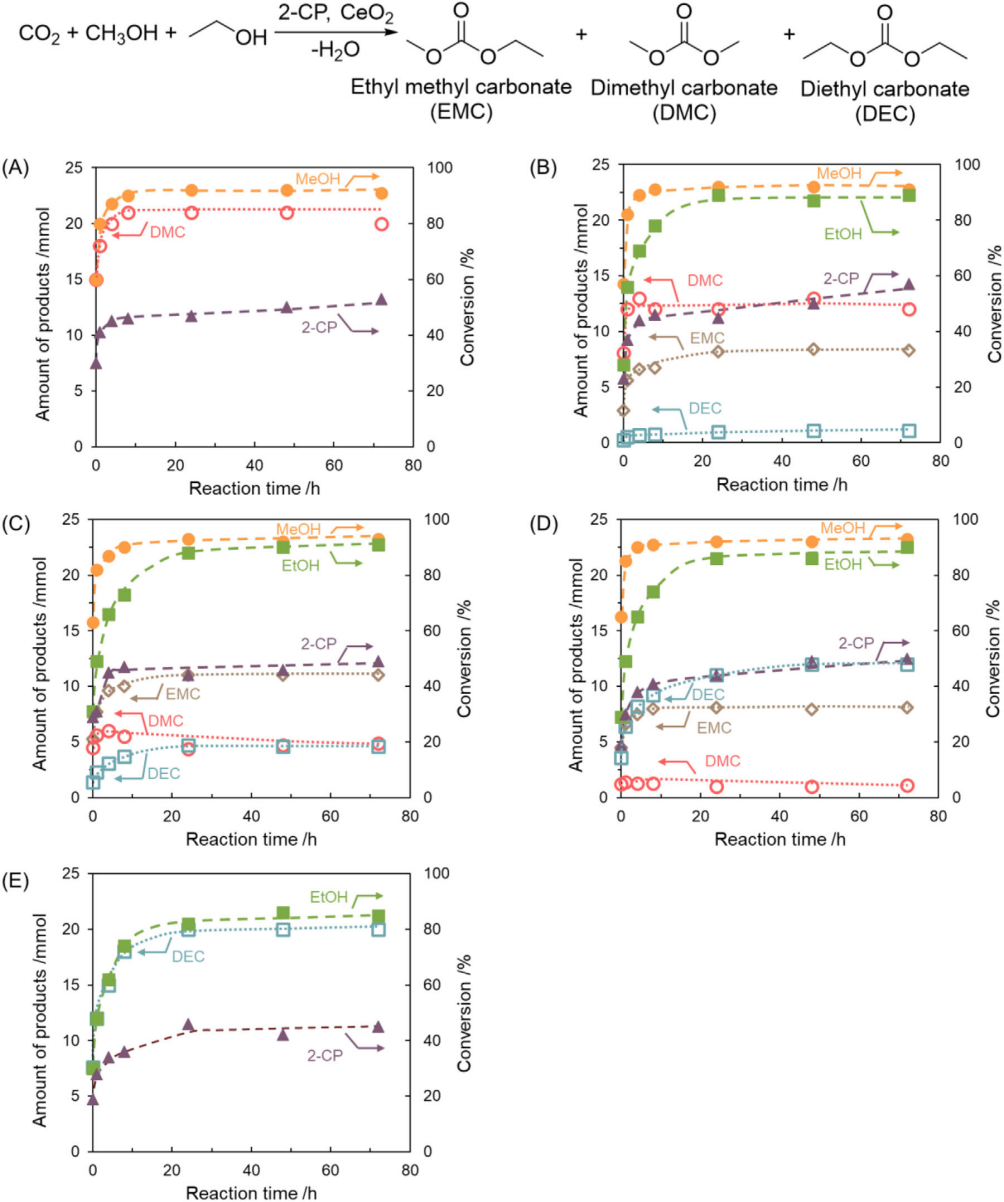
Time courses for synthesis of ethyl methyl carbonate (EMC) from MeOH, EtOH, and CO_2_ over CeO_2_ catalyst at the different MeOH/EtOH molar ratios: (A) 50/0; (B) 37.5/12.5; (C) 25:25; (D) 12.5/37.5; (E) 0/50. Reaction conditions: either or both of MeOH and EtOH 50 mmol in total; CeO_2_ 2.0 mmol; 2‐CP 50 mmol; acetonitrile 100 mmol; CO_2_ 5 MPa (r.t.); 393 K; 0–72 h. Detailed data are summarized in Table S5.



Figure 1 depicts the time courses of EMC synthesis at different molar ratios of MeOH to EtOH with their constant total amount (i.e., 50 mmol) in the presence of both CeO_2_ and 2‐cyanopyridine (2‐CP; 50 mmol) at 393 K, and the raw data are listed in Table S5. This loading of 2‐CP corresponds to twice the stoichiometric amount required for the production of organic carbonates from 50 mmol of the two alcohols. The control reactions with the sole use of either MeOH or EtOH produced dimethyl carbonate (DMC; Figure 1A) and diethyl carbonate (Figure 1E), respectively. Under the current reaction conditions, the highest production amounts were 21 mmol for DMC (corresponding to 84% yield based on methanol) and 20 mmol for diethyl carbonate (80% yield based on ethanol). These yields were lower than previously reported values achieved in the presence of 2‐CP at the same temperature, possibly because of the differences in the reaction scale and molar ratio of alcohol to 2‐CP [78, 98]. The higher reactivity of MeOH compared to EtOH [93] was demonstrated by the higher production amount of DMC at 0 h and steeper slope for the DMC production in Figure 1A than those for diethyl carbonate in Figure 1E. Upon the formation of such organic carbonates, almost stoichiometric amount of 2‐CP was hydrated into picolinamide (PA). Yet, the extension of reaction time made the undesired side reactions prominent, such as the reaction of PA and alcohol to give alkyl picolinate and NH_3_ (Equation 13) and subsequent reaction of NH_3_ with organic carbonate to provide alkyl carbamate (Equation 14), consistent with previous reports [78, 93, 98].

(13)

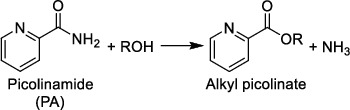






(14)

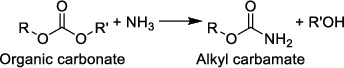




**FIGURE 2:**
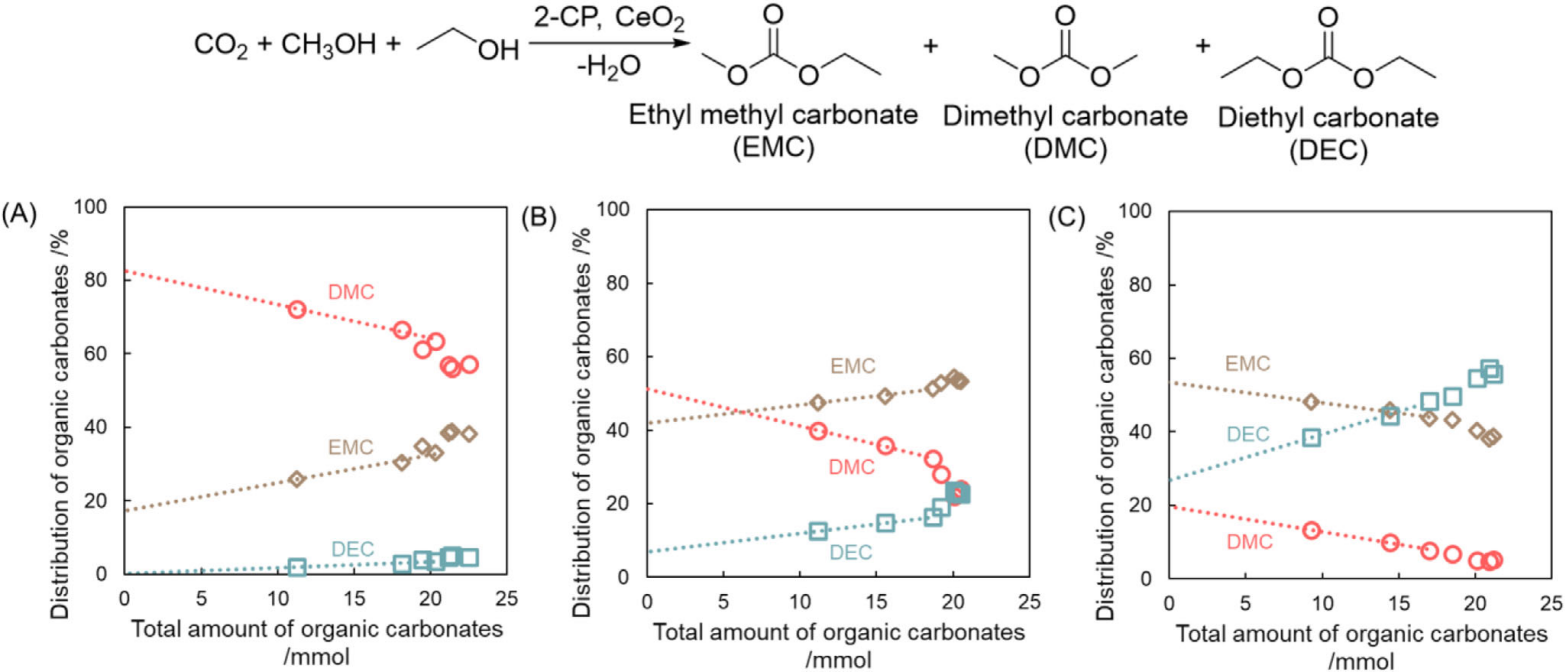
Parametric plot for the distribution of three organic carbonates as a function of their total amount at the different MeOH/EtOH molar ratios: (A) 37.5/12.5; (B) 25:25; (C) 12.5/37.5. The dotted lines in these graphs were generated by linear extrapolation using the three leftmost data points. Reaction conditions: either or both of MeOH and EtOH 50 mmol in total; CeO_2_ 2.0 mmol; 2‐CP 50 mmol; acetonitrile 100 mmol; CO_2_ 5 MPa (r.t.); 393 K; 0–72 h. Detailed data are summarized in Table S5.

The reactions operated in the presence of both MeOH and EtOH produced the mixture of three organic carbonates (i.e., EMC, DMC, and diethyl carbonate), and their distribution was dependent on the initial molar ratio of MeOH/EtOH. At the equimolar ratio (i.e., MeOH/EtOH = 25/25; Figure 1C), the major organic carbonate product was EMC at all reaction times ranging from 0 to 72 h. The amount of EMC reached 11 mmol at 24 h, which was more than twice as high as those of DMC (4.4 mmol) and diethyl carbonate (4.7 mmol); the distribution of EMC among these three organic carbonates was calculated to be 54%. At 72 h that was assumed to be enough long time to provide the equilibrated composition of the three organic carbonates, the distributions of EMC, DMC, and diethyl carbonate were 53%, 24%, and 23%, respectively (Figure 2B and Table S5). These distributions agreed well with those calculated simply from the probability of formation of each organic carbonate on the basis of mathematical combination considering the initial amounts of MeOH and EtOH (the calculated distributions of EMC, DMC, and diethyl carbonate were 50%, 25%, and 25%, respectively; detailed calculation procedure is available in the Supporting Information). This good agreement suggested that the equilibrium composition was controlled by such probability because of the similar standard enthalpies of reaction (Δ_r_ *H*°) with respect to the syntheses of each organic carbonate from CO_2_ and alcohol(s) (i. –17.1 kJ mol^−1^ for EMC [99, 100, 101], –18.0 kJ mol^−1^ for DMC [99, 101, 102], and –16.0 kJ mol^−1^ for diethyl carbonate [99, 101, 103]; Tables S6 and S7) as well as their transesterification (Tables S6 and S7).

Both the formation amount and distribution of EMC under the equimolar condition were higher than those provided under the MeOH‐excess and EtOH‐excess conditions (Figure 1B,D). DMC was the second dominant product in this reaction; its amount gradually increased from 0 to 4 h but then slightly decreased upon the further extension of reaction time. The distribution of DMC among the three organic carbonates (i.e., EMC, DMC, and diethyl carbonate) gradually decreased at longer reaction times (Table S5); instead, the distribution of EMC increased.

Figure 2 displays the parametric plots for the distribution of the three organic carbonates as a function of their total amount, the latter of which corresponds to the reaction time. The linear extrapolation based on the three leftmost data points toward 0 mmol of total amount of organic carbonates reflects the distribution of the three organic carbonates within a kinetic regime, thereby providing insights into the relative ease of formation of each organic carbonate as well as reaction pathways. The current extrapolation offers two indications. The first indication arises from the highest distribution of DMC among the three organic carbonates at 0 mmol of the total amount of organic carbonates; its distribution gradually decreases upon the increase in the total amount of organic carbonates in contrast to the increases of distributions of EMC and diethyl carbonate. These trends provide the possibility of DMC as an intermediate for the other two organic carbonates. Given that transesterification using organic carbonates is typically accelerated by strong acid or base catalysts [21, 25, 28, 38, 39, 43] as well as that both acidity and basicity of CeO_2_ are weak [104], strong base sites generated at the interface between CeO_2_ and 2‐CP, which were demonstrated experimentally and theoretically [105, 106], may behave as catalytically active sites for this transesterification step. Another indication originates from the high distribution of EMC even at the beginning of reaction. This high distribution of EMC can be rationalized by the contribution of direct reaction of MeOH, EtOH, and CO_2_ to form EMC. These data thus posit to the involvement of two reaction pathways in the formation of EMC from the mixture of MeOH, EtOH, and CO_2_: (i) the indirect pathway consisting of DMC production from MeOH and CO_2_ (i.e., homo‐carboxylation) and subsequent transesterification between DMC and EtOH, and (ii) the direct pathway from MeOH, EtOH, and CO_2_ (i.e., cross‐carboxylation). The involvement of the DMC‐mediated indirect pathway was also supported by control experiments using DMC and EtOH as substances at 393 K (Figure S3 and Table S8), where the rapid transesterification between DMC and EtOH to form EMC was observed regardless of the presence/absence of 2‐CP. These reaction data indicated that once DMC is generated in the reaction mixture in the synthesis of EMC from MeOH, the produced DMC readily participated in the subsequent transesterification with EtOH to produce EMC.

In the reactions to synthesize EMC from MeOH, EtOH, and CO_2_ introduced above, 2‐CP was selectively converted into PA upon its hydration. However, the extension of reaction times led to the formation of undesired byproducts consisting of methyl picolinate, ethyl picolinate, methyl carbamate, and ethyl carbamate (Table S5). Therefore, too long reaction time was unfavorable in terms of side reactions, consistent with the synthesis of DMC and diethyl carbonate (Figure 1A,E). When a reduced amount of 2‐CP (i.e., 25 mmol), which is the stoichiometric amount for producing organic carbonates from 50 mmol of the two alcohols, was employed, the amounts of EMC, DMC, and diethyl carbonate formed became lower than those obtained under conditions using 50 mmol of 2‐CP (Figure S4 and Table S9). The conversions of MeOH, EtOH, and 2‐CP reached *ca*. 80% but did not exceed 90%. In contrast, the use of 50 mmol of 2‐CP enabled the conversion of MeOH to exceed 90%. Although a lower amount of 2‐CP is desirable from the viewpoint of isolating the target compound from the reaction mixture, reaction conditions employing a higher amount of 2‐CP (e.g., 50 mmol) are favorable for driving the reaction forward.

Different from the case at the equimolar MeOH/EtOH ratio, the reactions at the MeOH/EtOH ratio of 37.5/12.5 (i.e., MeOH excess over EtOH) produced DMC as a major product (Figure 1B). The amount of DMC steeply increased within 1 h and became constant at 12–13 mmol. Due to the high reactivity of MeOH, the distribution of DMC among the three organic carbonates at the beginning was estimated to be over 80% (Figure 2A). Along with DMC, EMC was formed gradually, and its amount became unchanged at 8.2–8.4 mmol after 24 h, which was lower than the amount achieved under the conditions at the equimolar ratio of MeOH/EtOH (i.e., 11 mmol, Figure 1C). The distribution of EMC in the three organic carbonates (i.e., EMC, DMC, and diethyl carbonate) was below 40% due to the prominent formation of DMC under the current conditions. Since a large portion of EtOH was used for the formation of EMC, the amount of diethyl carbonate was quite low at the beginning and was at most 1.1 mmol even at 24 h or longer reaction times regardless of *ca*. 90% conversion of EtOH. The distributions of EMC, DMC, and diethyl carbonate at 72 h were 39%, 56%, and 5%, respectively (Figure 2A and Table S5). Consistent with the case of the equimolar condition (*vide supra*), these contributions matched well with the probability‐controlled composition (i.e., EMC/DMC/diethyl carbonate = 37.5/56.25/6.25% in the Supporting Information). This consistency posited to the probability‐basis composition of these three organic carbonates at the equilibrium.

As shown in Figure 1D, the reactions operated at the MeOH/EtOH ratio of 12.5/37.5 (i.e., EtOH excess over MeOH) provided diethyl carbonate mainly, followed by EMC. The amounts of diethyl carbonate and EMC increased upon the extension of reaction time to 24 h and became plateau. Although the reactivity of EtOH is lower compared to MeOH, the excess amount of EtOH over MeOH (i.e., high concentration of EtOH) led to the dominant production of diethyl carbonate even at the beginning of the reaction (Figure 2C). In this reaction condition, the distribution of EMC was initially the highest, while that of DMC was low (∼20%), in contrast to the reaction conditions introduced above. The distributions of both EMC and DMC gradually decreased upon the extension of reaction time, while that of diethyl carbonate increased. Akin to the transesterification between DMC and ethanol to form EMC (*vide supra*), EMC was likely to be consumed by the intermolecular reaction with EtOH to produce diethyl carbonate, which is consistent with previous reports [10, 12, 21, 23, 31]. The distributions of EMC, DMC, and DEC at the equilibrium were 39%, 5%, and 56%, respectively (Figure 2C and Table S5), which matched well with the probability‐basis composition (i.e., EMC/DMC/diethyl carbonate = 37.5/6.25/56.25% in the Supporting Information). Therefore, the equilibrated distribution of EMC, DMC, and diethyl carbonate was underscored to be governed and calculable by the initial ratio of MeOH to EtOH. Altogether, the equimolar condition was preferable for the synthesis of EMC from MeOH, EtOH, and CO_2_ in terms of the highest amount and distribution of EMC.

We further investigated the reusability of CeO_2_ catalyst under the equimolar condition with the calcination of the spent catalyst at 873 K after each reaction cycle (see Experimental). The reason for the calcination treatment for the spent CeO_2_ catalyst after each run is due to the observation of organic deposits on the catalyst surfaces in the TG‐DTA profiles (Figure S5). It should also be noted that in this examination, the reaction data away from the equilibrium values were obtained intentionally by adjusting the reaction time to 0 h, to avoid the overestimation of catalyst stability [107]. The CeO_2_ catalyst kept its activity within at least four reaction cycles (Table S10). The BET specific surface area and crystallite structure were almost unchanged through this examination (Table S10 and Figure S6). These data, therefore, demonstrated the good reusability of CeO_2_ catalyst in the synthesis of EMC.

### 3.2 Synthesis of Methyl Propyl Carbonate (MPC)

**FIGURE 3:**
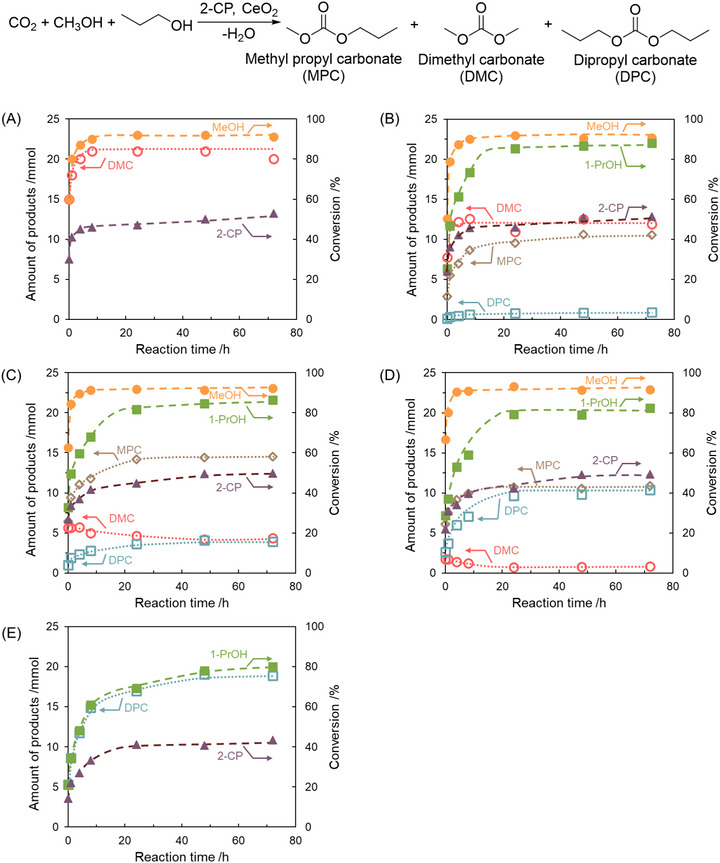
Time courses for synthesis of methyl propyl carbonate (MPC) from MeOH, 1‐PrOH, and CO_2_ over CeO_2_ catalyst at the different MeOH/1‐PrOH molar ratios: (A) 50/0; (B) 37.5/12.5; (C) 25:25; (D) 12.5/37.5; (E) 0/50. Reaction conditions: either or both of MeOH and 1‐PrOH 50 mmol in total; CeO_2_ 2.0 mmol; 2‐CP 50 mmol; acetonitrile 100 mmol; CO_2_ 5 MPa (r.t.); 393 K; 0–72 h. Detailed data are summarized in Table S11.



The next target is the reaction of methanol (MeOH), 1‐propanol (1‐PrOH), and CO_2_ to produce methyl propyl carbonate (MPC) (Equation 15). The reaction data for this reaction are shown in Figure 3 and Table S11.

(15)

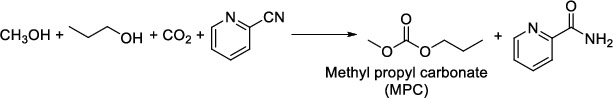




The sole use of 1‐PrOH provided dipropyl carbonate, and its maximum amount was 19 mmol, which corresponded to 76% yield based on 1‐PrOH (Figure 3E). The formation rate of dipropyl carbonate reflected by the reaction data at 0 h was 2.8‐fold lower than that of DMC (Figure 3A). As demonstrated previously, 2‐CP shows a slight steric effect against 1‐PrOH via competitive adsorption on the surfaces of CeO_2_ catalyst [93]. In addition to the lower reactivity of 1‐PrOH compared to MeOH, such steric effect of 2‐CP leads to the limitation of reaction field on CeO_2_ for activating substrate molecules, making the production of dipropyl carbonate more difficult than that of DMC.

In the same manner as the synthesis of EMC in Section 3.1, the reaction conditions at the different molar ratios of MeOH/1‐PrOH were adopted for the synthesis of MPC in the presence of 50 mmol of 2‐CP. The co‐presence of MeOH and 1‐PrOH enabled the one‐pot synthesis of MPC regardless of the molar ratios of MeOH/1‐PrOH. As shown in Figure 3C, the reaction under the equimolar condition yielded 15 mmol of MPC with 64% of its distribution among the three organic carbonates (DMC, MPC, and dipropyl carbonate); both values were higher than those under the MeOH‐excess and 1‐PrOH‐excess conditions (Figure 3B,D). Therefore, the equimolar condition was suitable for synthesizing MPC. The reactions with a reduced amount of 2‐CP (i.e., 25 mmol) led to a slight decrease in the amounts of MPC, DMC, and dipropyl carbonate (Figure S7 and Table S12), consistent with the synthesis of EMC (see Section 3.1).

**FIGURE 4:**
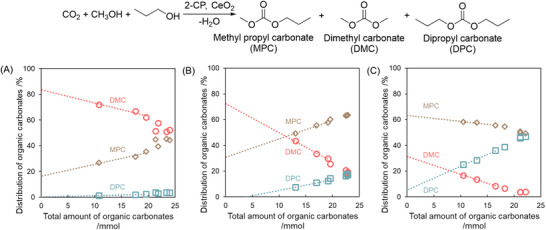
Parametric plot for the distribution of three organic carbonates as a function of their total amount at the different MeOH/1‐PrOH molar ratios: (A) 37.5/12.5; (B) 25:25; (C) 12.5/37.5. The dotted lines in these graphs were generated by linear extrapolation using the three leftmost data points. Reaction conditions: either or both of MeOH and 1‐PrOH 50 mmol in total; CeO_2_ 2.0 mmol; 2‐CP 50 mmol; acetonitrile 100 mmol; CO_2_ 5 MPa (r.t.); 393 K; 0–72 h. Detailed data are summarized in Table S11.

In the parametric plots for the distribution of the three organic carbonates versus their total amount (Figure 4), the extrapolation toward 0 mmol of total amount of the organic carbonates indicated the involvement of both direct and indirect pathways in the production of MPC—cross‐carboxylation of MeOH, 1‐PrOH, and CO_2_ and homo‐carboxylation of MeOH and CO_2_ followed by transesterification—consistent with the production of EMC (Section 3.1). Meanwhile, the extrapolated distribution of DMC in Figure 4B was slightly higher than that in Figure 2B, implying the higher contribution of the indirect pathway in the production of MPC possibly due to the lower reactivity of 1‐PrOH relative to ethanol. The same reason was invoked for the slightly higher distribution of DMC even under the 1‐PrOH‐excess condition in Figure 4C, compared to Figure 2C. Under the 1‐PrOH‐excess condition, the distributions of both DMC and MPC decreased upon the increase of reaction time, while that of dipropyl carbonate increased instead. These behaviors indicated that the produced MPC subsequently underwent the transesterification with 1‐PrOH to form dipropyl carbonate. The MeOH‐excess condition offered the ease of DMC production to provide the high distribution of DMC over 80% owing to the high reactivity of MeOH (Figure 4A), consistent with the discussion for Figure 2A. Such dominantly produced DMC was then consumed via the transesterification with 1‐PrOH to form MPC.

At the reaction time of 72 h under the equimolar condition, the distributions of MPC, DMC, and dipropyl carbonate were 64%, 19%, and 17%, respectively, which were close to the probability‐basis distribution (i.e., MPC/DMC/dipropyl carbonate = 50/25/25%, in the Supporting Information). Even under the MeOH‐excess and 1‐PrOH‐excess conditions, the experimentally observed and probability‐basis distributions were similar to each other. These relationships were again rationalized by the similar Δ_r_ *H*° with respect to the syntheses of each organic carbonate from CO_2_ and alcohol(s) (i.e., –17.7 kJ mol^−1^ for MPC [99, 101, 102, 108], –18.0 kJ mol^−1^ for DMC [99, 101, 102], and –17.5 kJ mol^−1^ for dipropyl carbonate [99, 101, 102, 108]; Tables S6 and S7) and also their transesterification (Tables S6 and S7).

### 3.3 Synthesis of Isopropyl Methyl Carbonate (iPMC)

The third target product in this study is isopropyl methyl carbonate (iPMC), which is synthesized from methanol (MeOH), 2‐propanol (2‐PrOH), and CO_2_ (Equation 16).

(16)

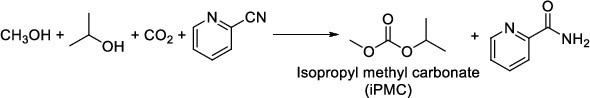




**FIGURE 5:**
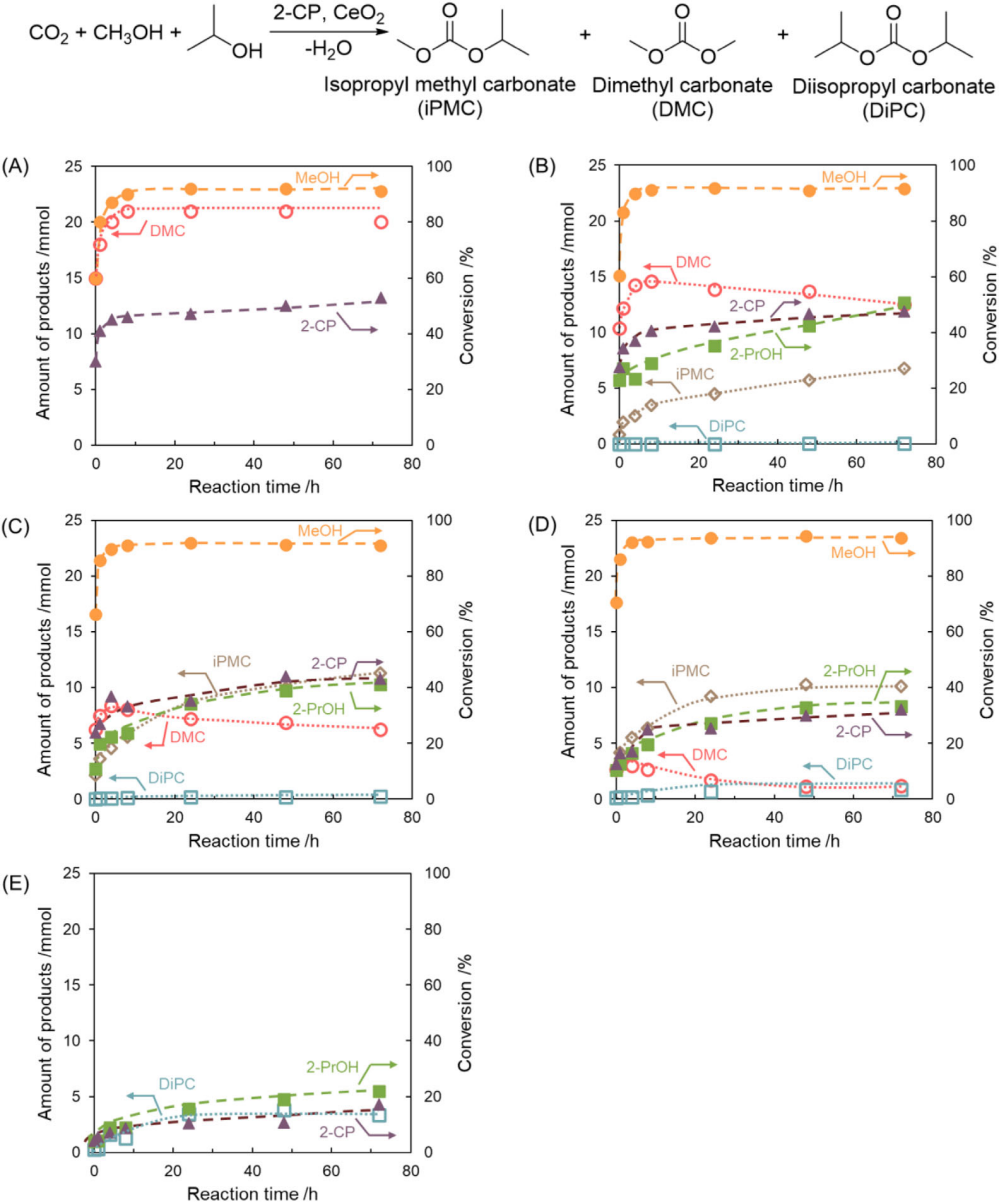
Time courses for synthesis of isopropyl methyl carbonate (iPMC) from MeOH, 2‐PrOH, and CO_2_ over CeO_2_ catalyst at the different MeOH/2‐PrOH molar ratios: (A) 50/0; (B) 37.5/12.5; (C) 25:25; (D) 12.5/37.5; (E) 0/50. Reaction conditions: either or both of MeOH and 2‐PrOH 50 mmol in total; CeO_2_ 2.0 mmol; 2‐CP 50 mmol; acetonitrile 100 mmol; CO_2_ 5 MPa (r.t.); 393 K; 0–72 h. Detailed data are summarized in Table S13.

The mixture of MeOH and 2‐PrOH in the presence of CeO_2_ and 2‐CP (50 mmol) successfully produced iPMC (Figure 5 and Table S13), consistent with the syntheses of asymmetric organic carbonates in Sections 3.1 and 3.2. The highest amounts of iPMC achieved here were 10–11 mmol under the equimolar and 2‐PrOH‐excess conditions. Meanwhile, the distribution of iPMC within the three organic carbonates (i.e., iPMC, DMC, and diisopropyl carbonate) exceeded 80% at 48 h and did not decrease even at longer reaction time of 72 h under the 2‐PrOH‐excess condition. This distribution of iPMC was clearly higher than that under the equimolar condition (64%). In addition, the significant discrepancy was observed between the experimentally determined distributions of the three organic carbonates (e.g., iPMC/DMC/diisopropyl carbonate = 83/10/7% under the 2‐PrOH‐excess condition; Table S13) and probability‐basis distributions under the identical condition (iPMC/DMC/diisopropyl carbonate = 37.5/6.25/56.25% in the Supporting Information). These observations were different from the syntheses of ethyl methyl carbonate (EMC; Sections 3.1) and methyl propyl carbonate (MPC; Sections 3.2) discussed above, where the equimolar condition was optimum for maximizing the distribution of asymmetric organic carbonates. Such discrepancies originated from the low reactivity of 2‐PrOH due to its bulky alkyl moiety and severely limited reaction field on the CeO_2_ surfaces by the adspecies of 2‐CP (Figure S8) [93], resulting in the quite low formation amount of diisopropyl carbonate and high distribution of iPMC. Indeed, in the reactions operated with the sole substrate of 2‐PrOH, the amount of diisopropyl carbonate was at most 3.8 mmol, which corresponded to only 15% yield on the basis of 2‐PrOH (Figure 5E), positing the difficulty in the synthesis of diisopropyl carbonate. Such difficulty in the production of symmetric organic carbonates thus offers the opportunity for the selective synthesis of asymmetric organic carbonate from the mixture of MeOH and 2‐PrOH.

The reactions in the presence of 25 mmol of 2‐CP provided almost the consistent amounts of iPMC, DMC, and diisopropyl carbonate with those with 50 mmol of 2‐CP (Figure S9 and Table S14). This consistency is different from the cases of the syntheses of the other asymmetric carbonates, where the conditions using 50 mmol of 2‐CP provided the slightly higher amounts of three organic carbonates compared to those using 25 mmol of 2‐CP (see above and below). Given that *ca*. 40% of 2‐CP still remained in the reaction mixture, the difficulty in the formation of iPMC (and diisopropyl carbonate) originating from steric hindrance given by both bulky 2‐PrOH and 2‐CP adspecies on CeO_2_ (*vide supra*) could lead to the consistency between the two different 2‐CP loadings.

**FIGURE 6:**
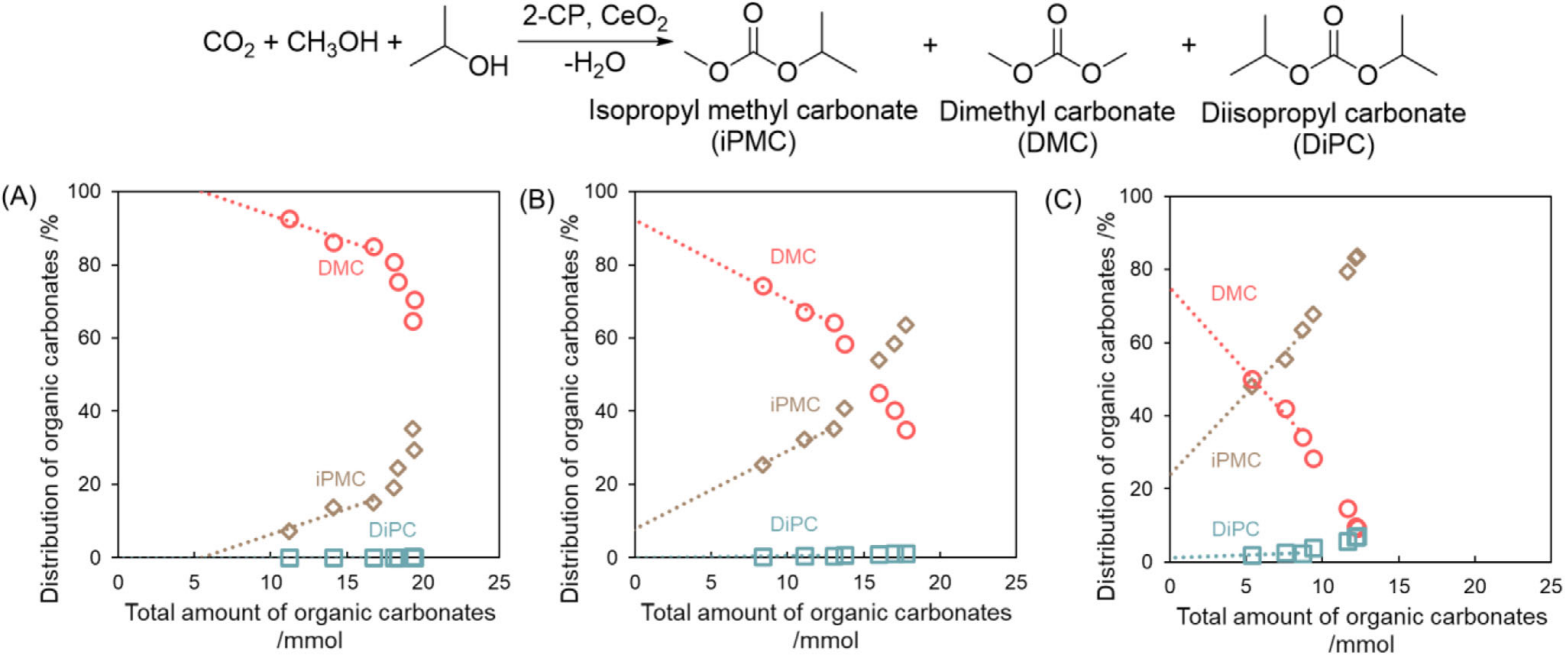
Parametric plot for the distribution of three organic carbonates as a function of their total amount at the different MeOH/2‐PrOH molar ratios: (A) 37.5/12.5; (B) 25:25; (C) 12.5/37.5. The dotted lines in these graphs were generated by linear extrapolation using the three leftmost data points. Reaction conditions: either or both of MeOH and 2‐PrOH 50 mmol in total; CeO_2_ 2.0 mmol; 2‐CP 50 mmol; acetonitrile 100 mmol; CO_2_ 5 MPa (r.t.); 393 K; 0–72 h. Detailed data are summarized in Table S13.

To compare the syntheses of iPMC and other asymmetric organic carbonates, the distributions of iPMC, DMC, and diisopropyl carbonate under the equimolar condition were plotted against their total amount in Figure 6. Under the equimolar condition (Figure 6B), the extrapolated distribution of DMC at 0 mmol of total amount of the three organic carbonates was >90%, which was clearly higher than those observed for the production of other asymmetric organic carbonates (<70%; see Figures 2B and 4B). The distribution of DMC gradually decreased upon the reaction progress, while that of iPMC increased instead. Likewise, DMC was suggested to be the dominant product under the MeOH‐excess condition (Figure 6A). Even in the 2‐PrOH‐excess condition, the initial distribution of DMC was quite high at the beginning of the reactions (Figure 6C), in stark contrast to the cases of other asymmetric organic carbonates where the distribution of asymmetric organic carbonate was higher than that of DMC (Figures 2C and 4C). These data indicated that the production of iPMC proceeded dominantly through the indirect pathway consisting of homo‐carboxylation between MeOH and CO_2_ followed by transesterification between DMC and 2‐PrOH. The high contribution of the indirect pathway to the production of iPMC is different from the involvement of both direct and indirect pathways in those of other asymmetric organic carbonates investigated in this study and can be rationalized by the quite low reactivity of 2‐PrOH (*vide supra*).

### 3.4 Synthesis of Butyl Methyl Carbonate (BMC)

The last asymmetric organic carbonate targeted in this study is butyl methyl carbonate (BMC) from methanol (MeOH), 1‐butanol (1‐BuOH), and CO_2_ (Equation 17).

(17)

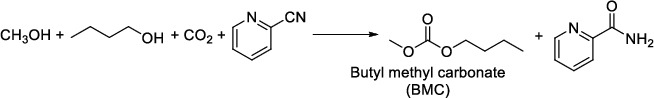




**FIGURE 7:**
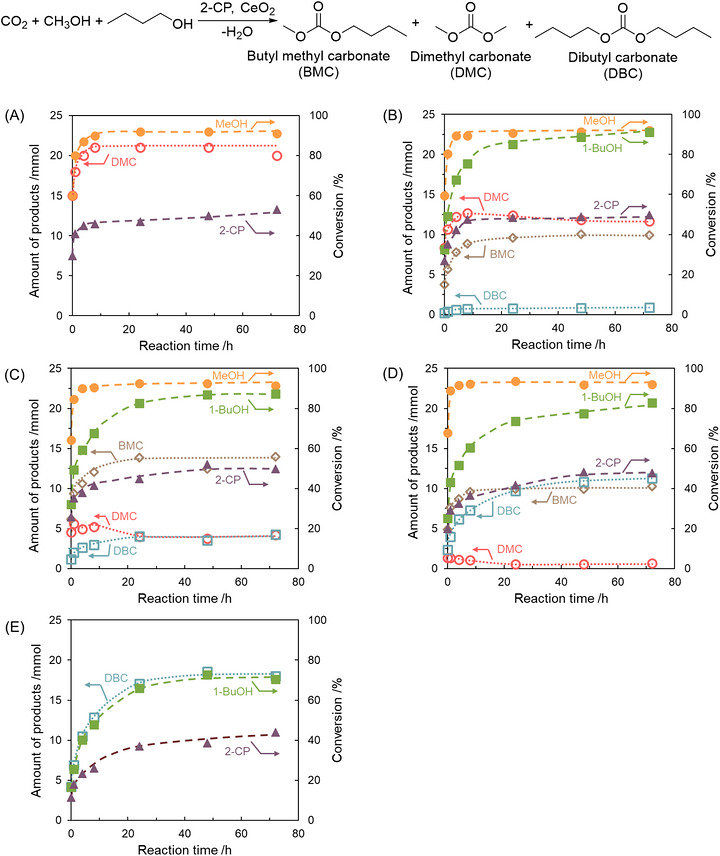
Time courses for synthesis of BMC from MeOH, 1‐BuOH, and CO_2_ over CeO_2_ catalyst at different molar ratio of MeOH/1‐BuOH: (A) 50/0; (B) 37.5/12.5; (C) 25:25; (D) 12.5/37.5; (E) 0/50. Reaction conditions: either or both of MeOH and 1‐BuOH 50 mmol in total; CeO_2_ 2.0 mmol; 2‐CP 50 mmol; acetonitrile 100 mmol; CO_2_ 5 MPa (r.t.); 393 K; 0–72 h. Detailed data are summarized in Table S15.

Figure 7 represents the time courses for the reactions of MeOH, 1‐BuOH, and CO_2_ in the presence of 2‐CP (50 mmol) over CeO_2_, and the detailed data are listed in Table S15. BMC was produced successfully from the mixture of MeOH, 1‐BuOH, and CO_2_ (Figure 7B–D). The highest formation amount of BMC (14 mmol) and distribution of BMC (63%) among BMC, DMC, and dibutyl carbonate were provided under the equimolar condition (Figure 7C). The optimum molar ratio of two alcohols that provided the highest amount and distribution of target asymmetric organic carbonate were consistent with those for ethyl methyl carbonate (EMC; Section 3.1) and methyl propyl carbonate (MPC; Section 3.2) while different from that for isopropyl methyl carbonate (iPMC; Section 3.3). As demonstrated in Figure 7E, the reactivity of 1‐BuOH was high enough to produce dibutyl carbonate with its amount up to 19 mmol, which corresponded to 76% yield based on 1‐BuOH. The 1‐BuOH‐excess condition, therefore, inevitably formed dibutyl carbonate along with BMC, resulting in the relatively low distribution of BMC, in contrast to the case of iPMC. The lower amount of 2‐CP resulted in the slightly lower amounts of BMC, DMC, and dibutyl carbonate (Figure S10 and Table S16), consistent with the production of EMC and MPC (*vide supra*).

**FIGURE 8:**
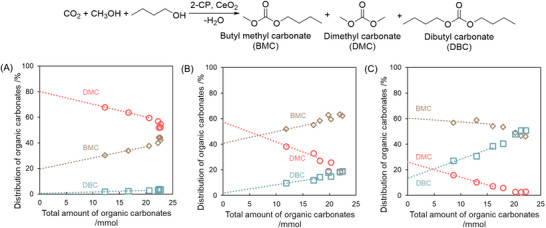
Parametric plot for the distribution of three organic carbonates as a function of their total amount at the different MeOH/1‐BuOH molar ratios: (A) 37.5/12.5; (B) 25:25; (C) 12.5/37.5. The dotted lines in these graphs were generated by linear extrapolation using the three leftmost data points. Reaction conditions: either or both of MeOH and 1‐BuOH 50 mmol in total; CeO_2_ 2.0 mmol; 2‐CP 50 mmol; acetonitrile 100 mmol; CO_2_ 5 MPa (r.t.); 393 K; 0–72 h. Detailed data are summarized in Table S15.

As shown in Figure 8, the extrapolated distributions of DMC and BMC at 0 mmol of the total amount of DMC, BMC, and dibutyl carbonate led to the same conclusions as the production of EMC (Section 3.1) and MPC (Section 3.2). Thus, the production of BMC was indicated to proceed through both the direct pathway (i.e., cross‐carboxylation of MeOH, 1‐BuOH, and CO_2_) and the indirect route (i.e., homo‐carboxylation of MeOH and CO_2_ and subsequent transesterification between DMC and 1‐BuOH).

The distributions of BMC, DMC, and dibutyl carbonate at 72 h under the equimolar condition were estimated to be 62%, 19%, and 19%, respectively. These values were close to those calculated from the probability for the formation of these three organic carbonates by considering the initial amounts of MeOH and 1‐BuOH (i.e., BMC/DMC/dibutyl carbonate = 50/25/25%, in the Supporting Information). Besides, such similarity was also found for the MeOH‐excess and 1‐BuOH‐excess conditions (Table S15). Although we lack thermodynamic data for the syntheses of BMC and dibutyl carbonate, akin to the discussion in Sections 3.1 and 3.2, the probability‐basis distribution observed here would be due to similar Δ_r_ *H*° values for the syntheses of each organic carbonate from CO_2_ and alcohol(s) as well as for the transesterification between the organic carbonate and alcohol.

### 3.5 Consistency and Difference Among Four Reaction Systems for Synthesizing Asymmetric Organic Carbonates

**FIGURE 9:**
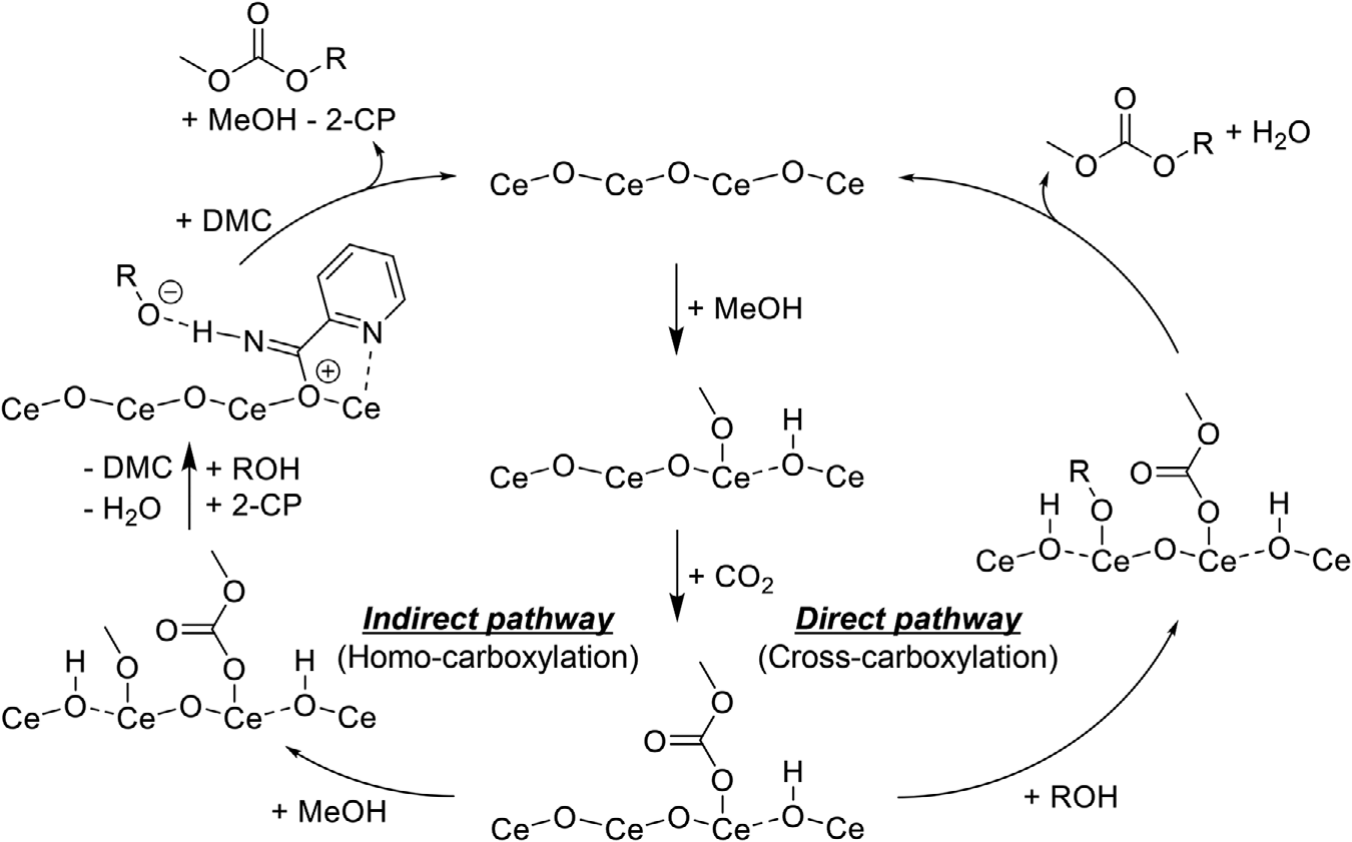
Plausible reaction mechanisms for one‐pot synthesis of asymmetric organic carbonate.

In all the four reaction systems for producing asymmetric organic carbonates introduced in the former sections, DMC was found to play a vital role as the intermediate, as illustrated in Figure 9. In the DMC‐mediated indirect route, methanol (MeOH) is chemically adsorbed onto CeO_2_ to generate methoxy adspecies, which subsequently reacts with CO_2_ to be methyl carbonate adspecies; all these steps are known to proceed readily, demonstrated by in situ Fourier transform infrared (FT‐IR) spectroscopy [50, 109, 110]. This methyl carbonate adspecies undergoes nucleophilic attack by methoxy group to produce DMC. As we proposed previously, these reaction steps for MeOH proceed smoothly over the surfaces of CeO_2_ catalyst even in the presence of 2‐cyanopyridine (2‐CP), while 2‐CP interacts tightly with CeO_2_ to limit the reaction field over CeO_2_ against long‐chain (≥C3) or bulky alcohols (Figure S8) [93]. In other words, 2‐CP is useful for the in situ removal of the co‐produced water in the synthesis of DMC from MeOH and CO_2_, enabling the equilibrium shift toward the product side; in contrast, the 2‐CP adspecies rather limits the progress of reactions using long‐chain (≥C3) or bulky alcohols. Owing to the positive role of 2‐CP as a dehydrating agent in the production of DMC, this organic carbonate is accumulated in a reaction medium and behaves as a transesterification agent that reacts with the other alcohol (ROH) to form corresponding alkyl methyl carbonate. The interfacial sites between CeO_2_ and 2‐CP adspecies that were previously demonstrated to exhibit strong basicity [105, 106] would contribute to such transesterification reaction. This reaction route via the production of DMC as the intermediate has been indicated to be involved in all the reactions investigated in this study, while the degree of its contribution is dependent on the reactivity of ROH used to produce asymmetric organic carbonate. In the cases of ethanol (EtOH), 1‐propanol (1‐PrOH), and 1‐butanol (1‐BuOH) as ROH, the direct cross‐carboxylation of MeOH, ROH, and CO_2_ proceeded (Figure 9) in parallel with the DMC‐mediated indirect pathway. In stark contrast, the low reactivity of 2‐propanol (2‐PrOH) made the cross‐carboxylation pathway difficult, and the DMC‐mediated indirect pathway became dominant.

The reactivity of ROH with longer alkyl chain (i.e., EtOH, 1‐PrOH, and 1‐BuOH) is lower than that of MeOH particularly in the presence of 2‐CP and PA due to the reason mentioned above [93]. Yet, the formation of corresponding symmetric organic carbonates still proceeded to a greater or less extent via either or both of direct route from ROH + CO_2_ and indirect route from ROH + organic carbonate (i.e., DMC or asymmetric carbonate). Judging from the time‐dependence of distribution (*vide supra*), asymmetric organic carbonates are more likely than DMC to be the intermediates for the formation of dialkyl carbonates other than DMC. As a result, the reactions of MeOH, ROH, and CO_2_ provide a mixture of three organic carbonates (i.e., alkyl methyl carbonate, DMC, and dialkyl carbonate). In the cases of EtOH, 1‐PrOH, and 1‐BuOH used as ROH, the MeOH‐excess and ROH‐excess conditions make the formation of DMC and dialkyl carbonate favorable, respectively, resulting in the relatively low distribution of asymmetric organic carbonate. The equimolar ratio of MeOH to ROH is, therefore, optimum. In stark contrast to the primary alcohols, the low reactivity of secondary alcohol 2‐PrOH, whose adsorption onto CeO_2_ is inhibited by the adspecies of 2‐CP and PA [93], enabled the selective synthesis of isopropyl methyl carbonate (iPMC, >80% distribution) because of the difficulty in the formation of diisopropyl carbonate. In this case, the 2‐PrOH‐excess condition was favorable for maximizing the distribution of iPMC.

The distributions of asymmetric alkyl methyl carbonate, DMC, and dialkyl carbonate at long reaction times were found to agree with the equilibrated compositions calculated from the probability of organic carbonate formation by considering the initial amounts of MeOH and ROH, except for the case of 2‐PrOH. Such consistency originated from the nearly the same enthalpies of reaction (Δ_r_ *H*°) among the direct production of organic carbonate from CO_2_ and alcohol(s) and those among the transesterification between organic carbonate and alcohols. In stark contrast, the bulky alkyl moiety of 2‐PrOH led to the mismatch with the probability‐basis distribution of iPMC, DMC, and diisopropyl carbonate.

## 4 Conclusions

This work examined the possibility of one‐pot synthesis of alkyl methyl carbonates using CO_2_, methanol (MeOH), and other alcohols (ROH) as reactants with a CeO_2_ catalyst and 2‐cyanopyridine dehydrant. All the tested alcohols in this study (i.e., ethanol, 1‐propanol, 2‐propanol, and 1‐butanol) with MeOH produced the corresponding alkyl methyl carbonates, yet the co‐production of DMC and dialkyl carbonate was inevitable. The distribution of these three organic carbonates was dominated by two factors: initial molar ratio of MeOH to ROH and reactivity of ROH. In the cases of ethanol, 1‐propanol, and 1‐butanol as ROH, the equimolar condition (i.e., MeOH/ROH molar ratio of 25:25) was optimum for producing alkyl methyl carbonate with the highest amount and distribution among three organic carbonates (i.e., alkyl methyl carbonate, DMC, and dialkyl carbonate). The use of primary alcohols as ROH inevitably generates corresponding dialkyl carbonates, and therefore, the ROH‐excess condition (i.e., MeOH/ROH molar ratio of 12.5/37.5) was unfavorable for maximizing the distribution of target alkyl methyl carbonates. In these reactions, two reaction pathways—(i) direct route composed of cross‐carboxylation of MeOH, ROH, and CO_2_ and (ii) indirect route consisting of homo‐carboxylation of MeOH and CO_2_ and subsequent transesterification between DMC and ROH—were suggested to proceed in parallel. In stark contrast to these reactions using primary alcohols, the low reactivity of 2‐propanol due to its bulky alkyl moiety impeded the production of diisopropyl carbonate even under the 2‐propanol‐excess condition, which was suitable for maximizing the amount and distribution (>80%) of isopropyl methyl carbonate. The low reactivity of 2‐propanol made the DMC‐mediated pathway dominant for producing the asymmetric organic carbonate. This work provides the first insights into the one‐pot cross‐carboxylation of MeOH and other alcohols with CO_2_ to form asymmetric organic carbonates using the combination of CeO_2_ and 2‐cyanopyridine and would serve as a basis for further studies that aim at improving product selectivity and synthesizing a broader range of asymmetric organic carbonates.

## Conflicts of Interest

The authors declare no conflicts of interest.

## Supporting information



Supporting Information is available online as PDF, including lists of previous reports, raw reaction data, detailed information about reagents and gas, data of control reactions, and GC‐MS data.

## Data Availability

The data supporting this article have been included as part of the Supplementary Information.
