# Supplementary material for: Cross‐Carboxylation of Methanol and Other Alcohols With CO2 Into Asymmetric Alkyl Methyl Carbonates Over a CeO2 Catalyst
Source: Chemistry. 2026 May 1;32(27):e71064. doi: 10.1002/chem.71064 (PMC13380397; doi:10.1002/chem.71064)
Supplement: Supplementary file 1 — Supporting Information is available online as PDF, including lists of previous reports, raw reaction data, detailed information about reagents and gas, data of control reactions, and GC‐MS data. [file CHEM-32-e71064-s001.pdf]

Supporting Information

Cross-Carboxylation of Methanol and Other Alcohols with CO_2 into Asymmetric Alkyl Methyl Carbonates over a CeO_2 Catalyst

Yuan Li,[a,b,†] Zechen Li,[a,†] Mizuho Yabushita,[a,*] Yoshinao Nakagawa,[a,‡] Keiichi Tomishige[a,c,*]

[a]*Department of Applied Chemistry, Graduate School of Engineering, Tohoku University, 6-6-07 Aoba, Aramaki, Aoba-ku, Sendai, Miyagi 980-8579, Japan*
[b]*Faculty of Environmental Science and Engineering, Kunming University of Science and Technology, Kunming, Yunnan 650500, China*
[c]*Advanced Institute for Materials Research (WPI-AIMR), Tohoku University, 2-1-1 Katahira, Aoba-ku, Sendai, Miyagi 980-8577, Japan*

[†]*These authors contributed to this work equally.*
[‡]*Current address: Division of Materials and Environment, Graduate School of Science and Technology, Gunma University, 1-5-1 Tenjin-cho, Kiryu, Gunma 376-8515, Japan*
**Corresponding authors: m.yabushita@tohoku.ac.jp (M.Y.); tomishige@tohoku.ac.jp (K.T.)*

[**Calculation for the probability of formation of each organic carbonate based on mathematical combination under MeOH-excess, equimolar, and ROH-excess conditions**]

In this work, we adjusted the total amount of methanol (MeOH) and the other alcohol (ROH) at the constant value of 50 mmol; therefore, the total combination is always calculated to be ${}_{(50N_A/1000)}C_2 = (10^{-6}N_A^2)\times50^2/2$ (N_A = Avogadro number). The number of the combination of different alcohols is calculated by the multiplication of the numbers of alcohol molecules. The distribution of each organic carbonate (D_X, where X represents organic carbonate) is thus calculated by the ratio of combinations as follows. In the following equations, alkyl methyl carbonate, dimethyl carbonate, and dialkyl carbonate are abbreviated as AMC, DMC, and DAC, respectively.

1. MeOH-excess condition (MeOH = 37.5 mmol; ROH = 12.5 mmol)

$$D_{AMC} = \frac{(37.5N_A/1000)\times(12.5N_A/1000)}{(10^{-6}N_A^2)\times50^2/2}\times100\% = 37.5\%$$

$$D_{DMC} = \frac{{}_{(37.5N_A/1000)}C_2}{(10^{-6}N_A^2)\times50^2/2}\times100\% = 56.25\%$$

$$D_{DAC} = \frac{{}_{(12.5N_A/1000)}C_2}{(10^{-6}N_A^2)\times50^2/2}\times100\% = 6.25\%$$

2. Equimolar condition (MeOH = 25 mmol; ROH = 25 mmol)

$$D_{AMC} = \frac{(25N_A/1000)\times(25N_A/1000)}{(10^{-6}N_A^2)\times50^2/2}\times100\% = 50\%$$

$$D_{DMC} = \frac{{}_{(25N_A/1000)}C_2}{(10^{-6}N_A^2)\times50^2/2}\times100\% = 25\%$$

$$D_{DAC} = \frac{{}_{(25N_A/1000)}C_2}{(10^{-6}N_A^2)\times50^2/2}\times100\% = 25\%$$

3. ROH-excess condition (MeOH = 12.5 mmol; ROH = 37.5 mmol)

$$D_{AMC} = \frac{(12.5N_A/1000)\times(37.5N_A/1000)}{(10^{-6}N_A^2)\times50^2/2}\times100\% = 37.5\%$$

$$D_{DMC} = \frac{{}_{(12.5N_A/1000)}C_2}{(10^{-6}N_A^2)\times50^2/2}\times100\% = 6.25\%$$

$$D_{DAC} = \frac{{}_{(37.5N_A/1000)}C_2}{(10^{-6}N_A^2)\times50^2/2}\times100\% = 56.25\%$$

Table S1. List of previous reports on the synthesis of alkyl methyl carbonates via transesterification between DMC and primary alcohols.[a]

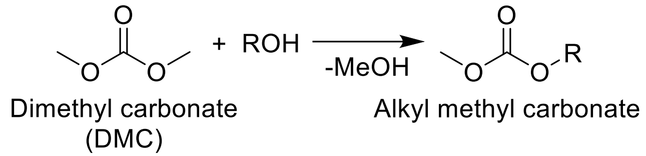

Entry	Substrates: DMC [mmol]	Substrates: Primary alcohol ([mmol])	Catalyst or promoter (amount)	Additive (amount)	Temp. [K]	Time [h]	Conv. [%] (Basis)	Yield [%] (Basis)	Ref.
Synthesis of ethyl methyl carbonate (EMC) from DMC and ethanol (EtOH)									
1[b]	–	EtOH	2.3 wt% MgO/HZSM-5 (320 mL)	–	363	–	99.4 (EtOH)	97.5 (EtOH)	S1
2[c]	–	EtOH	[CPIL-M]$_4$[PhO]	–	373	6	76.53 (EtOH)	69.02 (EtOH)	S2
3	200	EtOH (100)	[CPIL-Tr]$_1$[PhO] (0.68 g)	–	373	6	65.7 (EtOH)	57.0 (EtOH)	S3
4	30	EtOH (6)	MIL-101(AA) (55 mg)	–	Reflux	6	91.6 (EtOH)	75.4 (EtOH)	S4
5	75	EtOH (6)	MP-SO$_3$H-8 (0.3 mmol)	–	Reflux	24	95.3 (EtOH)	95.4 (EtOH)	S5
6	136.1	EtOH (11.3)	Novozym 435 (120 mg)	–	333	24	94.4 (EtOH)	90.3 (EtOH)	S6
7	6	EtOH (34)	$K_6Na_4[Ni(en)\{NiTa_{10}O_{32}\}]\cdot 22H_2O$ (1 μmol)	–	373	2	n.r.	60.2 (DMC)	S7
8	22	EtOH (65)	MOF-808 (50 mg)	–	363	24	86.3 (DMC)	49.8 (DMC)	S8
9	22	EtOH (65)	ZIF-67	–	363	24	63.8 (DMC)	54.5 (DMC)	S8
10	1110	EtOH (2170)	Lewatit K1221 (13.5 g)	–	348	26.7	54 (DMC)	53 (DMC)	S9
11	1110	EtOH (2170)	Nafion SAC-13 (13.5 g)	–	348	26.7	61 (DMC)	52 (DMC)	S9
12[d]	–	EtOH	[Emim]Im@SG-A12 (4 g)	–	353	–	49.0 (DMC) 60.3 (EtOH)	39.0 (DMC)	S10
13	30	EtOH (15)	14 wt% KATriz/Al$_2$O$_3$ (100 mg)	–	353	8	40 (DMC)	38 (DMC)	S11
14	60	EtOH (20)	0.3DBU-PO@ZIF-8 (0.27 g)	–	363	5	58.8 (EtOH)	55.7 (EtOH)	S12
15[e]	–	EtOH	Mg-Al mixed oxides	–	358	–	68.97	52.8	S13

			(7.6 g)				(EtOH)	(EtOH)	
16	10	EtOH (100)	Fe-Zn-1 (0.25 g)	–	443	8	97.4 (DMC)	38.1 (DMC)	S14
Synthesis of methyl propyl carbonate (MPC) from DMC and 1-propanol (1-PrOH)									
17	75	1-PrOH (6)	MP-SO_3H-8 (0.3 mmol)	–	Reflux	24	94.8 (1-PrOH)	94.8 (1-PrOH)	S5
18	10	1-PrOH (100)	Fe-Zn-1 (0.25 g)	–	443	8	93.4 (DMC)	60.8 (DMC)	S14
19	15	1-PrOH (5)	DBU (5 mmol)	CO_2 (1.0 MPa)	373	10	28.5 (DMC) 85.5 (1-PrOH)	82.9 (1-PrOH)	S15
20[f]	–	1-PrOH	TiO_2/Al_2O_3 (5 g)	–	403	30	62 (DMC)	51 (DMC)	S16
21	668	1-PrOH (134)	TBD (6.7 mmol)	–	Reflux	8	n.r.	45[g]	S17
22	17.8	1-PrOH (1)	Molecular sieves 4Å (0.5 g)	–	383	2	n.r.	99[g]	S18
Synthesis of butyl methyl carbonate (BMC) from DMC and 1-butanol (1-BuOH)									
23	30	1-BuOH (6)	MIL-101(AA) (89 mg)	–	Reflux	6	95.3 (1-BuOH)	57.0 (1-BuOH)	S4
24	75	1-BuOH (6)	MP-SO_3H-8 (0.3 mmol)	–	Reflux	24	100 (1-BuOH)	96.6 (1-BuOH)	S5
25	136.1	1-BuOH (11.3)	Novozym 435 (120 mg)	–	333	24	93.9 (1-BuOH)	87.3 (1-BuOH)	S6
26	10	1-BuOH (100)	Fe-Zn cyanide complex (0.25 g)	–	443	8	94.6 (DMC)	66.0 (DMC)	S14
27	15	1-BuOH (5)	DBU (5 mmol)	CO_2 (1.0 MPa)	373	10	27.7 (DMC) 83.1 (1-BuOH)	79.9 (1-BuOH)	S15
28	546	1-BuOH (109)	TBD (5.5 mmol)	–	Reflux	3	n.r.	85[f]	S17
29	17.8	1-BuOH (1)	Molecular sieves 4Å (0.5 g)	–	383	2	n.r.	99[f]	S18
30	5	1-BuOH (5)	[TMSPMI]Cl (1 mL)	–	353	4	n.r.	99 (n.r.)	S19
31	240.00	1-BuOH (6.00)	PTSA (0.30 mmol)	–	363	19	>99 (1-BuOH)	>99 (1-BuOH)	S20
32[h]	n.r.	1-BuOH (n.r.)	NaOMe (n.r.)	–	~363	1	100 (1-BuOH)	92 (1-BuOH)	S21
33[i]	n.r.	1-BuOH (n.r.)	MgO (n.r.)	–	363	1	98 (1-BuOH)	92 (1-BuOH)	S21

34	322	1-BuOH (13.5)	K_2CO_3/ZrO_2 (0.01 g)	–	403	0.5	36.4 (n.r.)	36.4 (n.r.)	S22
35	24	1-BuOH (1)	NZSM-5 (150 mg)	–	383	30	n.r.	91[f]	S23
36	45	1-BuOH (6)	CeO_2 (0.16 g)	–	363	12.5	98 (1-BuOH)	96 (1-BuOH)	S24

[a]Abbreviations: DMC = dimethyl carbonate; EtOH = ethanol; $[CPIL-M]_4[PhO]$ = imidazole-based ionic framework constructed from 1,3,5-tris(bromomethyl)benzene and biphenyldiimidazole; $[CPIL-Tr]_1[PhO]$ = phenolylated poly(4,4-bis(methyl-3-imidazol)biphenyl-1,3,5-tris(methyl)benzene); MIL-101(AA) = acetic acid-modified MIL-101; MP-SO_3H = sulfonated mesoporous polymer; en = ethylenediamine; n.r. = not reported; MOF = metal-organic framework; ZIF = zeolitic imidazolate framework; [Emim]Im = 1-ethyl-3-methylimidazole imidazolium; KATriz = 3-amino-1,2,4-triazole potassium; DBU = 1,8-diazabicyclic [5.4.0]undecarbon-7-ene; PO = phenol; Fe-Zn-1 = Fe^{2+}-Zn^{2+} cyanide complex; 1-PrOH = 1-propanol; TBD = 1,5,7-triazabicyclo[4.4.0]dec-5-ene; 1-BuOH = 1-butanol; [TMSPMI]Cl = 1-(3-trimethoxysilylpropyl)-3-methylimidazolium chloride; OMe = methoxide; NZSM-5 = nanocrystalline ZSM-5.

[b]Operated with a reactive distillation column; molar ratio of DMC/EtOH = 11/4; liquid hourly space velocity (LHSV) = 0.28 h^{-1}.

[c]Molar ratio of DMC/EtOH = 3/1; catalyst amount against DMC and EtOH = 3.00 wt%.

[d]Operated with a fixed-bed flow reactor; molar ratio of DMC/EtOH = 1/1; space velocity = 1 h^{-1}.

[e]Operated with a fixed-bed flow reactor; molar ratio of DMC/EtOH = 1/0.8; space velocity = 2.4 h^{-1}.

[f]Operated with a fixed-bed flow reactor; molar ratio of DMC/1-PrOH = 2/1; feeding rate of reaction mixture = 5 mL h^{-1}; gaseous hourly space velocity GHSV) = 360 h^{-1}; reaction pressure = 1.1 MPa.

[g]Isolated yield.

[h]Operated with a reactive vapor absorption system using molecular sieves; molar ratio of DMC/1-BuOH/NaOMe = 5:1:0.03.

[i]Operated with a reactive vapor absorption system using molecular sieves; molar ratio of DMC/1-BuOH = 5:1.

Table S2. List of previous reports on the synthesis of isopropyl methyl carbonate (iPMC) via transesterification between DMC and 2-propanol (2-PrOH).[a]

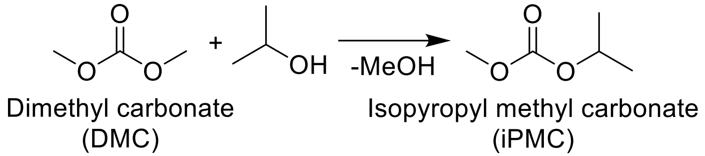

Entry	Substrates		Catalyst or promoter (amount)	Temp. [K]	Time [h]	Conv. [%] (Basis)	Yield [%] (Basis)	Ref.
	DMC [mmol]	2-PrOH [mmol]						
1	30	6	MIL-101(AA) (72 mg)	Reflux	6	49 (2-PrOH)	41 (2-PrOH)	S4
2	75	6	MP-SO_3H-8 (0.3 mmol)	Reflux	24	33.3 (2-PrOH)	33.3 (2-PrOH)	S5
3	654	131	TBD (6.5 mmol)	Reflux	3	n.r.	50[b] (n.r.)	S17

[a]Abbreviations: DMC = dimethyl carbonate; 2-PrOH = 2-propanol; MIL-101(AA) = acetic acid-modified MIL-101; MP-SO_3H = sulfonated mesoporous polymer; TBD = 1,5,7-triazabicyclo[4.4.0]dec-5-ene; n.r. = not reported.
[b]Isolated yield.

Table S3. List of previous reports on the synthesis of alkyl methyl carbonate from alkyl halide, methanol, and CO_2.[a]

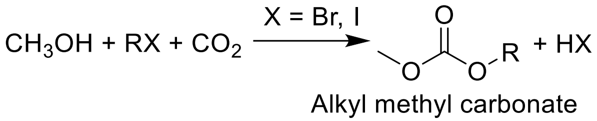

Entry	Substrates: CO_2 (amount)	Substrates: Alkyl sources (amount)	Catalyst or promoter (amount)	Solvent (amount)	Temp. [K]	Time [h]	Conv. [%] (Basis)	Yield [%] (Basis)	Ref.
Synthesis of ethyl methyl carbonate (EMC) from ethyl halide, methanol (MeOH), and CO_2									
1	CO_2 (0.25 MPa)	MeOH (2.0 mmol)<br>Bromoethane (1.0 mmol)	DBU<br>(1.5 mmol)	Acetonitrile<br>(0.5 mL)	343	2	n.r.	95<br>(n.r.)	S25
2	CO_2 (2 mmol[b])	MeOH (2 mmol)<br>Iodoethane (3.7 mmol)	$[C_4C_1Im][HCO_3]$<br>+ $C_4C_1Im–CO_2$<br>(2 mmol)	–	298	24	n.r.	27<br>(CO_2)	S26
Synthesis of methyl propyl carbonate (MPC) from n-propyl halide, methanol (MeOH), and CO_2									
3	CO_2 (0.25 MPa)	MeOH (2.0 mmol)<br>1-Bromopropane (1.0 mmol)	DBU<br>(1.5 mmol)	Acetonitrile<br>(0.5 mL)	343	2	n.r.	96<br>(1-Bromopropane)	S25
4	CO_2 (2 mmol[b])	MeOH (2 mmol)<br>1-Iodopropane (3.1 mmol)	$[C_4C_1Im][HCO_3]$<br>+ $C_4C_1Im–CO_2$<br>(2 mmol)	–	298	24	n.r.	29<br>(CO_2)	S26
Synthesis of isopropyl methyl carbonate (iPMC) from isopropyl halide, methanol (MeOH), and CO_2									
5	CO_2 (0.25 MPa)	MeOH (2.0 mmol)<br>2-Bromopropane (1.0 mmol)	DBU<br>(1.5 mmol)	Acetonitrile<br>(0.5 mL)	343	2	n.r.	78<br>(2-Bromopropane)	S25
6	CO_2 (2 mmol[b])	MeOH (2 mmol)<br>2-Iodopropane (3.0 mmol)	$[C_4C_1Im][HCO_3]$<br>+ $C_4C_1Im–CO_2$<br>(2 mmol)	–	298	24	n.r.	31<br>(CO_2)	S26
Synthesis of butyl methyl carbonate (BMC) from n-butyl halide, methanol (MeOH), and CO_2									
7	CO_2 (0.25 MPa)	MeOH (2.0 mmol)<br>1-Bromobutane (1.0 mmol)	DBU<br>(1.5 mmol)	Acetonitrile<br>(0.5 mL)	343	2	99<br>(1-Bromobutane)	97<br>(1-Bromobutane)	S25

[a]Abbreviation: MeOH = methanol; DBU = 1,8-diazabicyclo[5.4.0]-7-undecene; n.r. = not reported; $[C_4C_1Im][HCO_3]$ = 1-butyl-3-methylimidazolium hydrogen carbonate; $C_4C_1Im–CO_2$ = adduct of 1-butyl-3-methylimidazoline and CO_2; EtOH = ethanol.
[b]CO_2 was added into a reaction mixture as a form of adduct with C_4C_1Im.

Table S4. Detailed information about reagents and gas used in this study.

Reagent	Detailed information
CeO_2	HS grade, Daiichi Kigenso Kagaku Kogyo, used after calcination in air at 873 K for 3 h, S_{BET} = 86 m^2 g^{-1}
Methanol	FUJIFILM Wako Pure Chemical
Ethanol	Kanto Chemical
1-Propanol	Tokyo Chemical Industry
2-Propanol	FUJIFILM Wako Pure Chemical
1-Butanol	Tokyo Chemical Industry
2-Butanol	FUJIFILM Wako Pure Chemical
CO_2	99.995%, Taiyo Nippon Sanso
Acetonitrile	FUJIFILM Wako Pure Chemical
2-Cyanopyridine	Tokyo Chemical Industry
1-Hexanol	Tokyo Chemical Industry
Dimethyl carbamate	Tokyo Chemical Industry
Diethyl carbonate	FUJIFILM Wako Pure Chemical
Dipropyl carbonate	Sigma-Aldrich
Diisopropyl carbonate	BLD Pharmatech
Dibutyl carbonate	Tokyo Chemical Industry
Ethyl methyl carbonate	Tokyo Chemical Industry
Isopropyl methyl carbonate	Santa Cruz Biotechnology
Picolinamide	Tokyo Chemical Industry
Methyl picolinate	Tokyo Chemical Industry
Methyl carbamate	Tokyo Chemical Industry
Acetamide	Tokyo Chemical Industry

Table S5. Detailed data for Figure 1 (time courses for synthesis of ethyl methyl carbonate (EMC) from MeOH, EtOH, and CO_2 over CeO_2 catalyst at different molar ratio of MeOH/EtOH) and Figure 2 (parametric plot for the distribution of three organic carbonates as a function of their total amount at the different MeOH/EtOH molar ratios).[a,b]

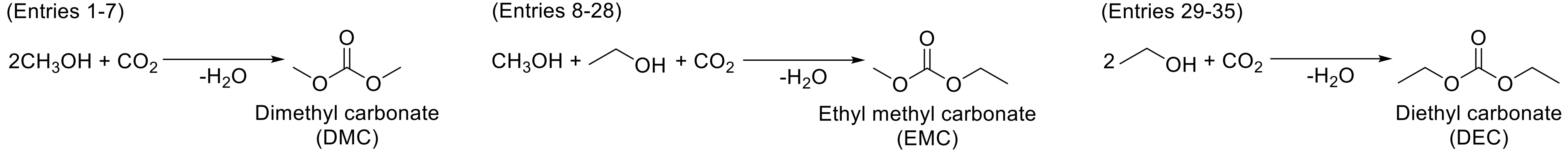

Entry	Alcohol [mmol]		*t*	Product amount [mmol]								Recovered reactant [mmol]			Conversion [%]			Distribution [%]			PA sel.	Balance [%]		
	MeOH	EtOH	[h]	EMC	DMC	DEC	PA	MP	EP	MC	EC	MeOH	EtOH	2-CP	MeOH	EtOH	2-CP	EMC	DMC	DEC	[%]	MeOH	EtOH	2-CP
1	50.0	–	0	–	15	–	15	0.10	–	0.00	–	20	–	36	60	–	29	–	100	–	>99	98	–	101
2			1	–	18	–	19	0.22	–	0.05	–	10	–	30	80	–	41	–	100	–	91	93	–	97
3			4	–	20	–	24	0.34	–	0.07	–	6.5	–	28	87	–	45	–	100	–	>99	95	–	103
4			8	–	21	–	24	0.51	–	0.17	–	4.8	–	27	90	–	46	–	100	–	>99	94	–	104
5			24	–	21	–	25	0.95	–	0.46	–	4.1	–	27	92	–	47	–	100	–	>99	94	–	105
6			48	–	21	–	22	1.4	–	0.79	–	4.1	–	25	92	–	50	–	100	–	88	96	–	97
7			72	–	20	–	22	2.4	–	1.8	–	4.3	–	24	91	–	53	–	100	–	83	97	–	96
8	37.5	12.5	0	2.9	8.1	0.23	12	0.04	<0.01	<0.01	<0.01	16	9.0	38	57	28	23	26	72	2	>99	93	99	101
9			1	5.6	12	0.54	20	0.14	0.03	<0.01	<0.01	6.8	5.6	31	82	56	37	30	67	3	>99	97	98	103
10			4	6.6	13	0.71	22	0.20	0.05	0.09	<0.01	4.3	3.8	28	89	69	44	33	63	4	99	97	95	100
11			8	6.7	12	0.76	22	0.27	0.08	0.11	<0.01	3.6	2.7	27	91	78	46	35	61	4	96	90	89	99
12			24	8.2	12	1.0	24	0.61	0.16	0.35	0.05	2.9	1.3	27	92	89	45	38	57	5	>99	97	93	103
13			48	8.4	13	1.1	25	0.80	0.22	0.44	0.11	3.0	1.6	25	92	87	50	38	57	5	99	100	99	101
14			72	8.3	12	1.1	27	1.1	0.28	0.66	0.19	3.3	1.3	21	91	89	57	39	56	5	95	99	98	100
15	25.0	25.0	0	5.3	4.5	1.4	13	0.05	0.02	<0.01	<0.01	9.4	17	37	63	31	29	48	40	13	89	94	102	97
16			1	7.7	5.6	2.3	17	0.06	0.04	<0.01	<0.01	4.6	13	34	82	49	31	49	36	15	>99	94	99	104
17			4	9.6	6.0	3.1	21	0.10	0.08	0.05	0.03	3.2	8.6	28	87	66	45	51	32	16	93	100	98	97
18			8	10	5.5	3.7	23	0.14	0.13	0.07	0.07	2.5	6.7	26	90	73	47	53	28	19	96	95	98	99
19			24	11	4.4	4.7	22	0.35	0.27	0.18	0.21	1.8	3.0	28	93	88	44	54	22	24	>99	86	93	101
20			48	11	4.7	4.6	22	0.55	0.40	0.31	0.32	1.9	2.6	27	93	90	46	54	23	23	96	92	93	100
21			72	11	4.9	4.6	23	0.74	0.56	0.45	0.46	1.9	2.3	26	93	91	49	53	24	23	95	93	93	100
22	12.5	37.5	0	4.5	1.2	3.6	11	0.01	0.02	<0.01	<0.01	4.4	27	41	65	29	19	48	13	39	>99	90	102	102
23			1	6.6	1.4	6.4	16	0.03	0.06	<0.01	<0.01	2.0	19	35	85	49	30	46	10	44	>99	89	103	101
24			4	7.5	1.3	8.2	19	0.04	0.10	<0.01	0.05	1.2	13	31	90	65	38	44	8	48	>99	90	99	101
25			8	8.0	1.3	9.2	20	0.06	0.17	<0.01	0.13	1.1	9.8	30	91	74	41	43	7	50	99	94	98	100
26			24	8.1	1.0	11	23	0.15	0.40	0.07	0.33	1.0	5.3	28	92	86	44	40	5	55	>99	88	96	103
27			48	7.9	1.0	12	25	0.24	0.60	0.24	0.52	1.0	5.3	26	92	86	49	38	5	57	>99	89	102	103
28			72	8.1	1.1	12	24	0.31	0.71	0.31	0.64	1.0	3.7	25	93	90	50	39	5	56	98	92	98	101
29	–	50.0	0	–	–	7.6	9.5	–	0.03	–	<0.01	–	36	41	–	30	19	–	–	100	>99	–	100	101
30			1	–	–	12	14	–	0.08	–	0.04	–	26	36	–	48	28	–	–	100	>99	–	102	100
31			4	–	–	15	17	–	0.14	–	0.09	–	19	33	–	62	34	–	–	100	>99	–	100	101
32			8	–	–	18	21	–	0.24	–	0.16	–	13	32	–	74	36	–	–	100	>99	–	99	106
33			24	–	–	20	22	–	0.56	–	0.43	–	8.9	27	–	82	46	–	–	100	97	–	98	100
34			48	–	–	20	24	–	0.77	–	0.62	–	7.1	29	–	86	42	–	–	100	>99	–	98	106
35			72	–	–	20	24	–	0.88	–	0.63	–	7.3	28	–	85	45	–	–	100	>99	–	97	106

[a]Reaction conditions: either or both of MeOH and EtOH 50 mmol in total; CeO_2 2.0 mmol; 2-CP 50 mmol; acetonitrile 100 mmol; CO_2 5.0 MPa (r.t.); 393 K; 0–72 h.

[b]Abbreviations in this table: MeOH = methanol; EtOH = ethanol; EMC = ethyl methyl carbonate; DMC = dimethyl carbonate; DEC = diethyl carbonate; PA = picolinamide; MP = methyl picolinate; EP = ethyl picolinate; MC = methyl carbamate; EC = ethyl carbamate; 2-CP = 2-cyanopyridine

Table S6. Enthalpy of formation ($\Delta_f H°$) for each compound in gas phase.

Entry	Compound	$\Delta_f H°$ [kJ mol^{-1}]	Ref.
1	Methanol (MeOH)	–200.7	S27
2	Ethanol (EtOH)	–235.1	S27
3	1-Propanol (1-PrOH)	–256.0	S28
4	Dimethyl carbonate (DMC)	–571.0	S29
5	Diethyl carbonate (DEC)	–637.9	S30
6	Ethyl methyl carbonate (EMC)	–604.5	S31
7	Dipropyl carbonate (DPC)	–681.1[a]	–
8	Methyl propyl carbonate (MPC)	–626.1[a]	–
9	CO_2	–393.5	S28
10	H_2O	–241.8	S28

[a]Calculated by ignoring the difference of temperatures for $\Delta_r H°$ (at 298.15 K) and $\Delta_r H$ of entries 8 and 9 in Table S7 (at 300 K)[S32].

Table S7. Enthalpy of reaction ($\Delta_r H°$) for synthesizing each organic carbonate, calculated from the thermodynamic data in Table S6.[a]

Entry	Reaction	$\Delta_r H°$ [kJ mol^{-1}]
Direct production of organic carbonate from CO_2 and alcohol(s)		
1	2MeOH + CO_2 → DMC + H_2O	–18.0
2	2EtOH + CO_2 → DEC + H_2O	–16.0
3	MeOH + EtOH → EMC + H_2O	–17.1
4	2 1-PrOH + CO_2 → DPC + H_2O	–17.5
5	MeOH + 1-PrOH → MPC + H_2O	–17.7
Transesterification between organic carbonate and alcohol		
4	DMC + 2EtOH → DEC + 2MeOH	+2.0
5	DMC + EtOH → EMC + MeOH	+0.9
6	DEC + MeOH → EMC + EtOH	–1.0
7	DMC + 2 1-PrOH → DPC + 2MeOH	+0.5[b]
8	DMC + 1-PrOH → MPC + MeOH	+0.3[c]
9	DPC + MeOH → MPC + 1-PrOH	–0.3[c]

[a]Abbreviations: MeOH = methanol; DMC = dimethyl carbonate; EtOH = ethanol; DEC = diethyl carbonate; EMC = ethyl methyl carbonate; 1-PrOH = 1-propanol; DPC = dipropyl carbonate; MPC = methyl propyl carbonate.

[b]Calculated by ignoring the difference of temperatures for $\Delta_r H°$ (at 298.15 K) and $\Delta_r H$ of entry 8 (at 300 K)[S32].

[c]$\Delta_r H$ at 300 K.[S32]

Table S8. Detailed data for Figure S3 (time courses for transesterification between DMC and EtOH to synthesize ethyl methyl carbonate (EMC) over CeO_2 (A and C) in the presence of 2-CP and (B and D) in the absence of 2-CP).[a,b]

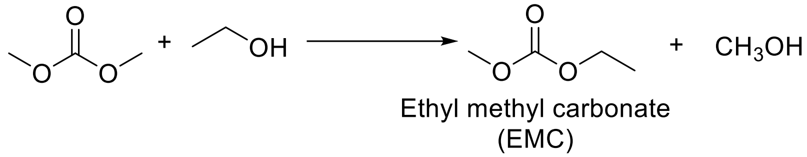

Entry	2-CP [mmol]	*t* [h]	Product amount [mmol]								Recovered reactant [mmol]			Conversion [%]			Distribution [%]			PA sel. [%]	Balance [%]		
			EMC	MeOH	DEC	PA	MP	EP	MC	EC	DMC	EtOH	2-CP	DMC	EtOH	2-CP	EMC	DMC	DEC		DMC	EtOH	2-CP
1	50	0	8.5	16	2.5	0.86	0.23	0.09	0.13	0.07	12	9.7	47	53	62	6	37	52	11	27	98	93	99
2		1	9.4	15	2.5	1.4	0.37	0.14	0.12	0.06	11	8.9	46	55	64	8	40	49	11	35	97	95	98
3		4	9.4	14	2.5	1.2	0.53	0.21	0.27	0.14	12	8.7	47	51	66	7	39	51	10	34	100	93	100
4		8	9.6	14	2.4	0.95	0.75	0.29	0.39	0.20	11	9.1	46	55	64	8	41	49	10	23	97	95	98
5		24	9.6	13	2.3	1.1	1.0	0.39	0.56	0.29	12	7.9	45	51	69	11	40	51	10	21	100	92	98
6		48	9.2	14	2.2	0.78	1.6	0.35	0.50	0.27	12	8.1	45	51	67	11	39	52	9	15	102	92	98
7		72	8.4	15	2.1	1.4	2.4	0.80	0.94	0.51	9.6	8.9	41	62	65	18	42	48	10	16	95	93	97
8	0	0	8.4	17	2.4	–	–	–	–	–	11	10	–	56	59	–	38	51	11	–	96	93	–
9		1	9.1	19	2.3	–	–	–	–	–	12	11	–	53	57	–	39	51	10	–	104	98	–
10		4	9.1	20	2.2	–	–	–	–	–	11	12	–	57	54	–	41	49	10	–	99	100	–
11		24	9.1	18	2.2	–	–	–	–	–	11	11	–	55	57	–	41	50	10	–	98	97	–

[a]Reaction conditions: DMC 25 mmol; EtOH 25 mmol; CeO_2 2.0 mmol; 2-CP 0 or 50 mmol; acetonitrile 100 mmol; Ar 5.0 MPa (r.t.); 393 K; 0–72 h.

[b]Abbreviations in this table: MeOH = methanol; EtOH = ethanol; EMC = ethyl methyl carbonate; DMC = dimethyl carbonate; DEC = diethyl carbonate; PA = picolinamide; MP = methyl picolinate; EP = ethyl picolinate; MC = methyl carbamate; EC = ethyl carbamate; 2-CP = 2-cyanopyridine.

Table S9. Detailed data for Figure S4 (time courses for synthesis of ethyl methyl carbonate (EMC) from MeOH, EtOH, and CO_2 over CeO_2 catalyst at the MeOH/EtOH molar ratio of 25:25 with the different amount of 2-CP).[a,b]

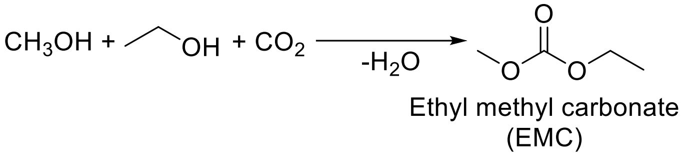

Entry	2-CP [mmol]	*t* [h]	Product amount [mmol]								Recovered reactant [mmol]			Conversion [%]			Distribution [%]			PA sel. [%]	Balance [%]		
			EMC	DMC	DEC	PA	MP	EP	MC	EC	MeOH	EtOH	2-CP	MeOH	EtOH	2-CP	EMC	DMC	DEC		MeOH	EtOH	2-CP
1	25	0	4.1	3.8	1.0	11	0.02	0.02	0.02	0.01	11	18	14	55	27	42	46	43	11	>99	91	97	103
2		1	6.8	5.1	2.1	16	0.08	0.05	0.06	0.03	5.5	13	9.0	78	48	64	49	36	15	98	91	96	100
3		4	8.2	5.3	2.7	18	0.17	0.10	0.11	0.08	4.0	9.6	6.9	84	62	72	51	33	17	>99	92	94	103
4		8	8.3	5.0	3.1	19	0.26	0.17	0.16	0.14	4.1	8.6	6.9	84	66	73	51	30	19	>99	91	93	104
5		24	9.2	4.9	3.7	20	0.46	0.42	0.32	0.30	3.7	5.7	5.1	85	77	80	52	28	21	>99	94	93	104
6		48	9.1	4.3	3.9	20	0.60	0.57	0.41	0.41	4.1	5.3	4.7	84	79	81	53	25	22	96	91	93	102
7		72	9.0	4.5	4.0	20	0.77	0.74	0.52	0.52	3.8	5.0	4.8	85	80	81	52	26	23	97	92	93	103
8	50	0	5.3	4.5	1.4	13	0.05	0.02	<0.01	<0.01	9.4	17	37	63	31	29	48	40	13	89	94	102	97
9		1	7.7	5.6	2.3	17	0.06	0.04	<0.01	<0.01	4.6	13	34	82	49	31	49	36	15	>99	94	99	104
10		4	9.6	6.0	3.1	21	0.10	0.08	0.05	0.03	3.2	8.6	28	87	66	45	51	32	16	93	100	98	97
11		8	10	5.5	3.7	23	0.14	0.13	0.07	0.07	2.5	6.7	26	90	73	47	53	28	19	96	95	98	99
12		24	11	4.4	4.7	22	0.35	0.27	0.18	0.21	1.8	3.0	28	93	88	44	54	22	24	>99	86	93	101
13		48	11	4.7	4.6	22	0.55	0.40	0.31	0.32	1.9	2.6	27	93	90	46	54	23	23	96	92	93	100
14		72	11	4.9	4.6	23	0.74	0.56	0.45	0.46	1.9	2.3	26	93	91	49	53	24	23	95	93	93	100

[a]Reaction conditions: MeOH 25 mmol; EtOH 25 mmol; CeO_2 2.0 mmol; 2-CP 25 or 50 mmol; acetonitrile 100 mmol; CO_2 5.0 MPa (r.t.); 393 K; 0–72 h.
[b]Abbreviations in this table: MeOH = methanol; EtOH = ethanol; EMC = ethyl methyl carbonate; DMC = dimethyl carbonate; DEC = diethyl carbonate; PA = picolinamide; MP = methyl picolinate; EP = ethyl picolinate; MC = methyl carbamate; EC = ethyl carbamate; 2-CP = 2-cyanopyridine.

Table S10. Reusability of CeO_2 catalyst in the synthesis of ethyl methyl carbonate (EMC) from MeOH, EtOH, and CO_2 with equimolar amount of MeOH to EtOH.[a,b]

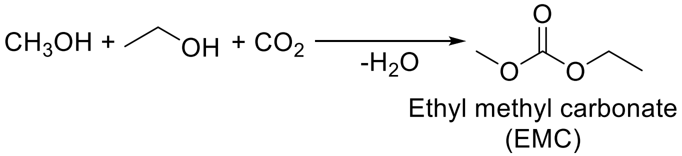

Entry	Run	S_{BET}[c] [m^2/g]	*t* [h]	Product amount [mmol]								Recovered reactant [mmol]			Conversion [%]			Distribution [%]			PA sel. [%]	Balance [%]		
				EMC	DMC	DEC	PA	MP	EP	MC	EC	MeOH	EtOH	2-CP	MeOH	EtOH	2-CP	EMC	DMC	DEC		MeOH	EtOH	2-CP
1	1st	83	0	5.4	4.7	1.5	14	0.02	0.02	0.02	0.01	8.5	15	36	67	39	28	47	41	13	99	91	95	100
2	2nd	75	0	5.2	4.8	1.5	13	0.02	0.02	0.02	0.01	8.0	16	37	68	38	27	45	42	13	97	91	95	99
3	3rd	76	0	5.5	5.0	1.6	14	0.02	0.02	0.02	0.01	7.9	15	36	69	39	28	46	41	13	98	93	96	99
4	4th	74	0	5.3	5.0	1.4	13	0.02	0.02	0.02	0.01	10	16	37	63	37	27	46	43	12	97	97	95	99

[a]Reaction conditions: MeOH 25 mmol; EtOH 25 mmol; CeO_2 2.0 mmol; 2-CP 50 mmol; acetonitrile 100 mmol; CO_2 5.0 MPa (r.t.); 393 K; 0 h. For each run, the spent catalyst was separated from the reaction mixture by centrifugation and decantation, dried at 333 K overnight, and calcined in air at 873 K for 3 h.
[b]Abbreviations in this table: MeOH = methanol; EtOH = ethanol; EMC = ethyl methyl carbonate; DMC = dimethyl carbonate; DEC = diethyl carbonate; PA = picolinamide; MP = methyl picolinate; EP = ethyl picolinate; MC = methyl carbamate; EC = ethyl carbamate; 2-CP = 2-cyanopyridine.
[c]Specific surface area.

Table S11. Detailed data for Figure 3 (time courses for synthesis of methyl propyl carbonate (MPC) from MeOH, 1-PrOH, and CO_2 over CeO_2 catalyst at different molar ratio of MeOH/1-PrOH) and Figure 4 (parametric plot for the distribution of three organic carbonates as a function of their total amount at the different MeOH/1-PrOH molar ratios).[a,b]

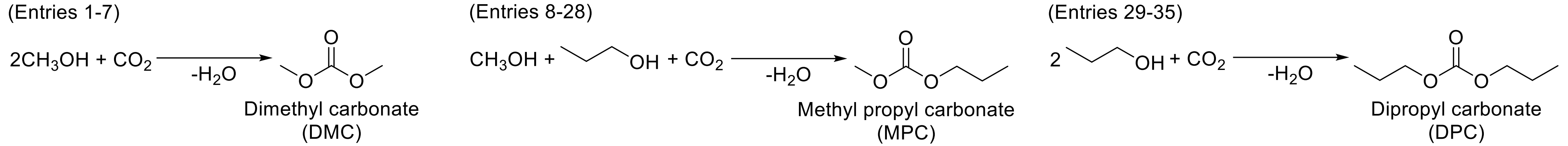

Entry	Alcohol [mmol]		*t*	Product amount [mmol]								Recovered reactant [mmol]			Conversion [%]			Distribution [%]			PA sel.	Balance [%]		
	MeOH	1-PrOH	[h]	MPC	DMC	DPC	PA	MP	PP	MC	PC	MeOH	1-PrOH	2-CP	MeOH	1-PrOH	2-CP	MPC	DMC	DPC	[%]	MeOH	1-PrOH	2-CP
1	50.0	–	0	–	15	–	15	0.10	–	0.00	–	20	–	36	60	–	29	–	100	–	>99	98	–	101
2			1	–	18	–	19	0.22	–	0.05	–	10	–	30	80	–	41	–	100	–	91	93	–	97
3			4	–	20	–	24	0.34	–	0.07	–	6.5	–	28	87	–	45	–	100	–	>99	95	–	103
4			8	–	21	–	24	0.51	–	0.17	–	4.8	–	27	90	–	46	–	100	–	>99	94	–	104
5			24	–	21	–	25	0.95	–	0.46	–	4.1	–	27	92	–	47	–	100	–	>99	94	–	105
6			48	–	21	–	22	1.3	–	0.79	–	4.1	–	25	92	–	50	–	100	–	88	96	–	97
7			72	–	20	–	22	2.4	–	1.8	–	4.3	–	24	91	–	53	–	100	–	83	97	–	96
8	37.5	12.5	0	2.9	7.8	0.15	11	0.03	<0.01	<0.01	<0.01	19	9.4	38	50	25	24	27	72	1	94	98	100	99
9			1	5.5	12	0.33	17	0.09	0.02	<0.01	<0.01	8.0	6.7	32	79	47	36	31	67	2	96	99	103	99
10			4	7.0	12	0.47	20	0.19	0.06	0.05	<0.01	4.7	4.8	29	87	61	42	36	62	2	95	97	102	98
11			8	8.6	13	0.64	21	0.28	0.11	0.13	0.03	3.7	3.3	27	90	73	46	40	58	3	94	101	107	98
12			24	9.6	11	0.79	22	0.52	0.19	0.29	0.08	3.0	1.8	27	92	85	46	45	51	4	95	94	105	99
13			48	11	13	0.84	21	0.78	0.26	0.52	0.15	3.6	1.7	24	91	87	51	44	52	4	84	107	115	94
14			72	11	12	0.89	22	1.1	0.34	0.71	0.22	3.5	1.5	24	91	88	52	45	51	4	84	106	114	95
15	25.0	25.0	0	6.4	5.7	0.97	13	0.04	0.02	<0.01	<0.01	11	17	37	63	33	27	49	43	7	95	100	101	99
16			1	9.4	5.7	1.9	16	0.07	0.05	<0.01	<0.01	3.9	13	33	84	50	34	56	33	11	96	99	104	99
17			4	11	5.7	2.4	18	0.09	0.10	<0.01	0.02	2.7	10	32	89	60	37	58	30	12	96	100	104	99
18			8	12	5.0	2.8	20	0.12	0.16	0.05	0.06	2.2	8.1	29	91	68	42	60	25	14	96	96	102	99
19			24	14	4.6	3.6	21	0.25	0.29	0.14	0.16	2.1	4.6	28	92	82	45	63	21	16	94	102	106	98
20			48	14	4.2	4.1	22	0.43	0.48	0.27	0.32	2.2	3.9	25	91	85	50	63	19	18	88	103	109	96
21			72	15	4.4	3.9	22	0.52	0.61	0.35	0.43	2.0	3.4	25	92	86	50	64	19	17	90	103	108	97
22	12.5	37.5	0	6.1	1.8	2.6	11	0.02	0.04	<0.01	<0.01	4.7	27	41	67	29	22	58	17	25	96	102	102	99
23			1	7.5	1.8	3.7	16	0.03	0.07	<0.01	<0.01	2.6	24	35	80	37	31	58	14	29	>99	105	103	100
24			4	9.2	1.4	6.0	17	0.03	0.12	<0.01	<0.01	1.2	18	33	90	53	34	55	8	36	99	106	103	100
25			8	9.9	1.2	7.1	17	0.04	0.17	<0.01	0.11	1.2	15	30	91	59	40	55	7	39	88	106	106	95
26			24	11	0.75	9.6	21	0.10	0.40	<0.01	0.31	0.89	7.7	29	93	79	43	51	4	46	99	102	103	101
27			48	11	0.76	9.8	23	0.15	0.57	0.06	0.45	1.1	7.9	25	91	79	49	50	4	47	92	104	104	97
28			72	11	0.84	10	22	0.19	0.71	0.09	0.57	1.1	6.6	25	92	82	49	49	4	47	89	106	105	96
29	–	50.0	0	–	–	5.3	6.8	–	0.03	–	<0.01	–	40	43	–	21	14	–	–	100	96	–	100	99
30			1	–	–	8.6	10	–	0.06	–	0.04	–	33	39	–	34	22	–	–	100	92	–	100	98
31			4	–	–	12	13	–	0.12	–	0.098	–	26	36	–	48	27	–	–	100	99	–	100	100
32			8	–	–	15	16	–	0.14	–	0.12	–	20	33	–	61	33	–	–	100	93	–	99	98
33			24	–	–	17	18	–	0.31	–	0.28	–	15	29	–	69	41	–	–	100	89	–	100	96
34			48	–	–	19	22	–	0.64	–	0.52	–	11	30	–	78	41	–	–	100	>99	–	101	105
35			72	–	–	19	22	–	0.77	–	0.65	–	10	28	–	80	43	–	–	100	>99	–	98	102

[a]Reaction conditions: either or both of MeOH and 1-PrOH 50 mmol in total; CeO_2 2.0 mmol; 2-CP 50 mmol; acetonitrile 100 mmol; CO_2 5.0 MPa (r.t.); 393 K; 0–72 h.

[b]Abbreviations in this table: MeOH = methanol; 1-PrOH = 1-propanol; MPC = methyl propyl carbonate; DMC = dimethyl carbonate; DPC = dipropyl carbonate; PA = picolinamide; MP = methyl picolinate; PP = propyl picolinate; MC = methyl carbamate; PC = propyl carbamate; 2-CP = 2-cyanopyridine

Table S12. Detailed data for Figure S7 (time courses for synthesis of methyl propyl carbonate (MPC) from MeOH, 1-PrOH, and CO_2 over CeO_2 catalyst at the MeOH/1-PrOH molar ratio of 25:25 with the different amount of 2-CP).[a,b]

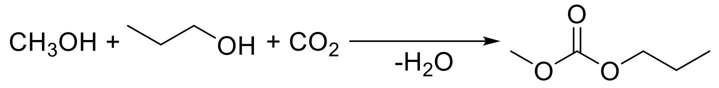

Methyl propyl carbonate (MPC)

Entry	2-CP [mmol]	*t* [h]	Product amount [mmol] MPC	DMC	DPC	PA	MP	PP	MC	PC	Recovered reactant [mmol] MeOH	1-PrOH	2-CP	Conversion [%] MeOH	1-PrOH	2-CP	Distribution [%] MPC	DMC	DPC	PA sel. [%]	Balance [%] MeOH	1-PrOH	2-CP
1	25	0	5.2	3.8	0.87	11	0.02	0.02	0.02	0.01	10	18	14	60	27	44	53	38	9	>99	92	100	100
2		1	8.0	5.0	1.5	15	0.05	0.06	0.06	0.03	4.9	14	10	80	43	61	55	34	10	>99	92	102	100
3		4	10	4.7	2.2	18	0.07	0.16	0.10	0.07	3.1	11	7.3	88	57	71	60	28	13	>99	91	101	101
4		8	11	4.0	2.7	18	0.11	0.25	0.14	0.13	2.8	8.7	6.6	89	65	73	63	22	15	>99	88	102	101
5		24	12	3.6	3.1	19	0.25	0.47	0.27	0.28	3.1	6.6	5.3	88	74	79	64	19	16	97	93	102	101
6		48	12	3.3	3.4	19	0.42	0.71	0.40	0.46	3.2	5.9	4.8	87	77	81	64	18	18	95	91	103	100
7		72	12	3.7	3.4	20	0.58	0.85	0.51	0.57	3.3	5.1	4.5	87	79	82	64	19	17	96	95	103	103
8	50	0	6.4	5.7	0.97	13	0.04	0.02	<0.01	<0.01	11	17	37	63	33	27	49	43	7	95	100	101	99
9		1	9.4	5.7	1.9	16	0.07	0.05	<0.01	<0.01	3.9	13	33	84	50	34	56	33	11	96	99	104	99
10		4	11	5.7	2.4	18	0.09	0.10	<0.01	0.02	2.7	10	32	89	60	37	58	30	12	96	100	104	99
11		8	12	5.0	2.8	20	0.12	0.16	0.05	0.06	2.2	8.1	29	91	68	42	60	25	14	96	96	102	99
12		24	14	4.6	3.6	21	0.25	0.29	0.14	0.16	2.1	4.6	28	92	82	45	63	21	16	94	102	106	98
13		48	14	4.2	4.1	22	0.43	0.48	0.27	0.32	2.2	3.9	25	91	85	50	63	19	18	88	103	109	96
14		72	15	4.4	3.9	22	0.52	0.61	0.35	0.43	2.0	3.4	25	92	86	50	64	19	17	90	103	108	97

[a]Reaction conditions: MeOH 25 mmol; 1-PrOH 25 mmol; CeO_2 2.0 mmol; 2-CP 25 or 50 mmol; acetonitrile 100 mmol; CO_2 5.0 MPa (r.t.); 393 K; 0–72 h.
[b]Abbreviations in this table: MeOH = methanol; 1-PrOH = 1-propanol; MPC = methyl propyl carbonate; DMC = dimethyl carbonate; DPC = dipropyl carbonate; PA = picolinamide; MP = methyl picolinate; PP = propyl picolinate; MC = methyl carbamate; PC = propyl carbamate; 2-CP = 2-cyanopyridine.

Table S13. Detailed data for Figure 5 (time courses for synthesis of isopropyl methyl carbonate (iPMC) from MeOH, 2-PrOH, and CO_2 over CeO_2 catalyst at different molar ratio of MeOH/2-PrOH) and Figure 6 (parametric plot for the distribution of three organic carbonates as a function of their total amount at the different MeOH/2-PrOH molar ratios).[a,b]

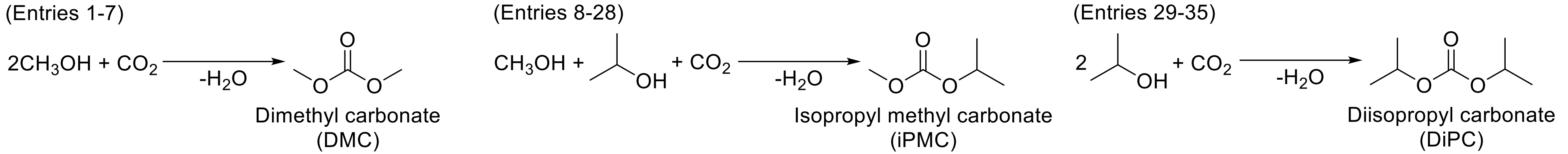

Entry	Alcohol [mmol]		*t*	Product amount [mmol]								Recovered reactant [mmol]			Conversion [%]			Distribution [%]			PA sel.	Balance [%]		
	MeOH	2-PrOH	[h]	iPMC	DMC	DiPC	PA	MP	iPP	MC	iPC	MeOH	2-PrOH	2-CP	MeOH	2-PrOH	2-CP	iPMC	DMC	DiPC	[%]	MeOH	2-PrOH	2-CP
1	50.0	–	0	–	15	–	15	0.10	–	<0.01	–	20	–	36	60	–	29	–	100	–	>99	98	–	101
2			1	–	18	–	19	0.22	–	0.05	–	10	–	30	80	–	41	–	100	–	91	93	–	97
3			4	–	20	–	24	0.34	–	0.07	–	6.5	–	28	87	–	45	–	100	–	>99	95	–	103
4			8	–	21	–	24	0.51	–	0.17	–	4.8	–	27	90	–	46	–	100	–	>99	94	–	104
5			24	–	21	–	25	0.95	–	0.46	–	4.1	–	27	92	–	47	–	100	–	>99	94	–	105
6			48	–	21	–	22	1.3	–	0.79	–	4.1	–	25	92	–	50	–	100	–	88	96	–	97
7			72	–	20	–	22	2.4	–	1.8	–	4.3	–	24	91	–	53	–	100	–	83	97	–	96
8	37.5	12.5	0	0.8	10	<0.01	12	0.08	<0.01	<0.01	<0.01	15	9.6	36	60	23	28	7	93	0	88	96	84	97
9			1	1.9	12	0.01	15	0.30	0.02	0.15	<0.01	6.3	9.2	33	83	27	34	14	86	0	86	88	89	96
10			4	2.5	14	0.01	17	0.26	0.02	0.09	<0.01	3.8	9.5	31	90	23	37	15	85	0	92	94	97	98
11			8	3.5	15	0.02	18	0.35	0.04	0.18	0.01	3.3	9.0	30	91	29	41	19	81	0	87	97	99	96
12			24	4.5	14	0.02	19	0.54	0.10	0.32	0.02	3.1	8.2	29	92	35	42	24	75	0	89	95	101	97
13			48	5.7	14	0.04	20	0.76	0.27	0.54	0.05	3.4	7.2	27	91	42	47	29	70	0	85	100	106	95
14			72	6.8	12	0.05	20	0.97	0.32	0.75	0.07	3.1	6.1	26	92	51	48	35	65	0	84	98	108	95
15	25.0	25.0	0	2.1	6.2	0.03	8.5	0.07	0.01	<0.01	<0.01	8.5	23	38	66	11	24	25	74	0	72	92	97	93
16			1	3.6	7.5	0.06	11	0.14	0.02	0.04	<0.01	3.7	20	36	86	20	27	32	67	1	85	88	95	96
17			4	4.6	8.4	0.08	12	0.16	0.05	0.09	<0.01	2.6	19	32	90	22	37	35	64	1	67	96	97	88
18			8	5.6	8.0	0.10	13	0.21	0.05	0.09	0.01	2.2	19	33	91	24	33	41	59	1	81	96	100	94
19			24	8.6	7.2	0.17	16	0.33	0.20	0.25	0.04	2.0	17	32	92	34	35	54	45	1	88	102	103	97
20			48	9.9	6.9	0.20	16	0.40	0.32	<0.01	0.07	2.2	15	28	91	39	44	58	40	1	73	104	104	90
21			72	11	6.2	0.23	19	0.51	0.48	<0.01	0.13	2.3	15	28	91	41	43	64	35	1	86	104	108	96
22	12.5	37.5	0	2.6	2.7	0.10	5.8	0.03	0.01	<0.01	<0.01	3.7	34	44	71	10	13	48	50	2	92	93	97	99
23			1	4.2	3.2	0.20	7.6	0.06	0.02	<0.01	<0.01	1.8	33	42	86	13	16	55	42	3	92	97	100	99
24			4	5.5	3.0	0.20	9.0	0.08	0.05	0.02	0.01	1.0	31	42	92	17	17	63	34	2	>99	98	99	101
25			8	6.3	2.7	0.38	9.8	0.09	0.10	0.06	0.02	1.0	30	38	92	20	25	68	28	4	79	101	100	95
26			24	9.2	1.7	0.68	10	0.11	0.26	0.10	0.08	0.79	27	37	94	27	25	79	15	6	81	108	102	96
27			48	10	1.2	0.87	11	0.11	0.40	0.14	0.15	0.73	25	35	94	33	30	83	9	7	75	106	101	94
28			72	10	1.2	0.84	11	0.11	0.52	0.15	0.23	0.80	25	34	94	33	32	83	10	7	68	109	100	91
29	–	50.0	0	–	–	0.28	1.5	–	<0.01	–	0.01	–	48	48	–	4	4	–	–	100	70	–	97	99
30			1	–	–	0.30	2.6	–	0.04	–	0.02	–	48	47	–	4	5	–	–	100	95	–	97	100
31			4	–	–	1.6	2.1	–	0.04	–	0.03	–	46	47	–	9	7	–	–	100	59	–	98	97
32			8	–	–	1.3	2.6	–	0.07	–	0.05	–	46	46	–	9	9	–	–	100	60	–	96	97
33			24	–	–	3.4	3.8	–	0.10	–	0.09	–	42	45	–	16	10	–	–	100	72	–	98	97
34			48	–	–	3.8	4.3	–	0.17	–	0.16	–	40	45	–	19	11	–	–	100	79	–	97	98
35			72	–	–	3.4	4.2	–	0.19	–	0.26	–	39	42	–	22	17	–	–	100	48	–	92	91

[a]Reaction conditions: either or both of MeOH and 2-PrOH 50 mmol in total; CeO_2 2.0 mmol; 2-CP 50 mmol; acetonitrile 100 mmol; CO_2 5.0 MPa (r.t.); 393 K; 0–72 h.

[b]Abbreviations in this table: MeOH = methanol; 2-PrOH = 2-propanol; iPMC = isopropyl methyl carbonate; DMC = dimethyl carbonate; DiPC = diisopropyl carbonate; PA = picolinamide; MP = methyl picolinate; iPP = isopropyl picolinate; MC = methyl carbamate; iPC = isopropyl carbamate; 2-CP = 2-cyanopyridine.

Table S14. Detailed data for Figure S9 (time courses for synthesis of isopropyl methyl carbonate (iPMC) from MeOH, 2-PrOH, and CO_2 over CeO_2 catalyst at the MeOH/2-PrOH molar ratio of 25:25 with the different amount of 2-CP:).[a,b]

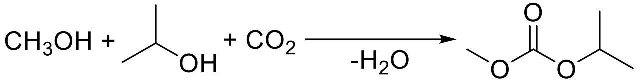

Isopropyl methyl carbonate
(iPMC)

Entry	2-CP [mmol]	*t* [h]	Product amount [mmol] iPMC	DMC	DiPC	PA	MP	iPP	MC	iPC	Recovered reactant [mmol] MeOH	2-PrOH	2-CP	Conversion [%] MeOH	2-PrOH	2-CP	Distribution [%] iPMC	DMC	DiPC	PA sel. [%]	Balance [%] MeOH	2-PrOH	2-CP
1	25	0	1.8	6.2	0.02	8.7	0.06	<0.01	0.04	<0.01	7.9	23	17	70	9	33	23	77	0	>99	88	98	102
2		1	2.9	7.8	0.03	11	0.11	<0.01	0.08	<0.01	3.9	22	14	85	13	44	27	72	0	>99	90	99	101
3		4	4.5	7.7	0.05	12	0.22	<0.01	0.14	0.01	2.4	21	13	90	18	49	37	63	0	99	91	100	100
4		8	5.4	7.5	0.07	13	0.17	0.11	0.19	0.02	2.1	20	12	92	21	51	42	58	1	98	91	101	100
5		24	7.4	6.5	0.10	13	0.25	0.25	0.32	0.04	2.1	18	11	92	30	56	53	46	1	94	91	101	99
6		48	8.7	5.4	0.13	13	0.26	0.49	0.43	0.08	2.0	16	11	92	36	56	61	38	1	93	88	102	99
7		72	9.2	5.8	0.14	14	0.31	0.71	0.56	0.13	2.2	15	10	91	39	59	61	38	1	92	95	103	99
8	50	0	2.1	6.2	0.03	8.5	0.07	0.01	<0.01	<0.01	8.5	23	38	66	11	24	25	74	0	72	92	97	93
9		1	3.6	7.5	0.06	11	0.14	0.02	0.04	<0.01	3.7	20	36	86	20	27	32	67	1	85	88	95	96
10		4	4.6	8.4	0.08	12	0.16	0.05	0.09	<0.01	2.6	19	32	90	22	37	35	64	1	67	96	97	88
11		8	5.6	8.0	0.10	13	0.21	0.05	0.09	0.01	2.2	19	33	91	24	33	41	59	1	81	96	100	94
12		24	8.6	7.2	0.17	16	0.33	0.20	0.25	0.04	2.0	17	32	92	34	35	54	45	1	88	102	103	97
13		48	9.9	6.9	0.20	16	0.40	0.32	<0.01	0.07	2.2	15	28	91	39	44	58	40	1	73	104	104	90
14		72	11	6.2	0.23	19	0.51	0.48	<0.01	0.13	2.3	15	28	91	41	43	64	35	1	86	104	108	96

[a]Reaction conditions: MeOH 25 mmol; 2-PrOH 25 mmol; CeO_2 2.0 mmol; 2-CP 25 or 50 mmol; acetonitrile 100 mmol; CO_2 5.0 MPa (r.t.); 393 K; 0–72 h.

[b]Abbreviations in this table: MeOH = methanol; 2-PrOH = 2-propanol; iPMC = isopropyl methyl carbonate; DMC = dimethyl carbonate; DiPC = diisopropyl carbonate; PA = picolinamide; MP = methyl picolinate; iPP = isopropyl picolinate; MC = methyl carbamate; iPC = isopropyl carbamate; 2-CP = 2-cyanopyridine.

Table S15. Detailed data for Figure 7 (time courses for synthesis of butyl methyl carbonate (BMC) from MeOH, 1-BuOH, and CO_2 over CeO_2 catalyst at different molar ratio of MeOH/1-BuOH) and Figure 8 (parametric plot for the distribution of three organic carbonates as a function of their total amount at the different MeOH/1-BuOH molar ratios).[a,b]

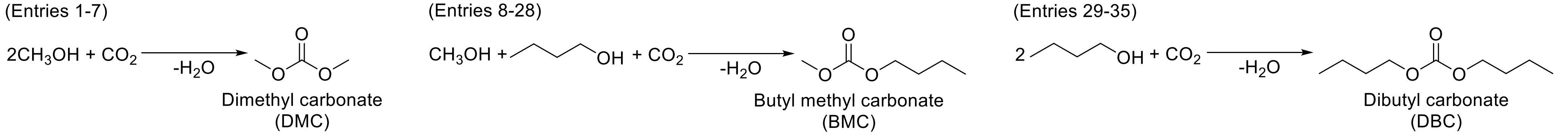

Entry	Alcohol [mmol]		*t*	Product amount [mmol]								Recovered reactant [mmol]			Conversion [%]			Distribution [%]			PA sel.	Balance [%]		
	MeOH	1-BuOH	[h]	BMC	DMC	DBC	PA	MP	BP	MC	BC	MeOH	1-BuOH	2-CP	MeOH	1-BuOH	2-CP	BMC	DMC	DBC	[%]	MeOH	1-BuOH	2-CP
1	50.0	–	0	–	15	–	15	0.10	–	<0.01	–	20	–	36	60	–	29	–	100	–	>99	98	–	101
2			1	–	18	–	19	0.22	–	0.05	–	10	–	30	80	–	41	–	100	–	91	93	–	97
3			4	–	20	–	24	0.34	–	0.07	–	6.5	–	28	87	–	45	–	100	–	>99	95	–	103
4			8	–	21	–	24	0.51	–	0.17	–	4.8	–	27	90	–	46	–	100	–	>99	94	–	104
5			24	–	21	–	25	0.95	–	0.46	–	4.1	–	27	92	–	47	–	100	–	>99	94	–	105
6			48	–	21	–	22	1.3	–	0.79	–	4.1	–	25	92	–	50	–	100	–	88	96	–	97
7			72	–	20	–	22	2.4	–	1.8	–	4.3	–	24	91	–	53	–	100	–	83	97	–	96
8	37.5	12.5	0	3.7	8.3	0.23	13	0.02	0.01	<0.01	<0.01	15	8.4	37	60	33	27	30	68	2	98	94	101	99
9			1	5.6	11	0.39	17	0.05	0.02	<0.01	<0.01	7.5	6.3	33	80	49	35	34	64	2	97	91	103	99
10			4	7.8	12	0.58	20	0.10	0.07	<0.01	<0.01	4.1	4.1	29	89	67	42	38	59	3	95	96	105	98
11			8	8.9	13	0.69	21	0.17	0.11	0.15	<0.01	4.0	3.1	26	89	75	47	40	57	3	90	103	108	96
12			24	9.6	12	0.76	22	0.31	0.17	0.26	0.06	3.5	1.9	26	91	85	48	42	55	3	92	102	106	97
13			48	10	12	0.85	23	0.60	0.25	0.46	0.09	3.3	1.4	26	91	89	49	44	52	4	92	100	108	98
14			72	9.9	12	0.86	23	0.77	0.29	0.62	0.13	3.0	1.1	25	92	91	50	44	52	4	90	100	105	97
15	25.0	25.0	0	6.2	4.6	1.2	12	0.02	0.02	<0.01	<0.01	9.1	17	37	64	32	26	52	38	10	94	96	102	98
16			1	9.4	5.6	2.0	17	0.03	0.05	<0.01	0.01	3.9	13	32	85	49	35	55	33	12	95	97	104	98
17			4	11	4.9	2.6	18	0.04	0.09	<0.01	0.03	2.5	10	31	90	59	38	58	27	14	94	92	105	98
18			8	12	5.2	3.0	20	0.05	0.14	0.05	0.06	2.4	8.1	29	90	68	41	59	26	15	95	100	105	98
19			24	14	4.0	4.1	21	0.15	0.33	0.04	0.13	1.9	4.4	28	93	83	45	63	18	19	92	96	107	97
20			48	12	3.8	3.5	21	0.25	0.23	0.24	0.25	1.9	3.3	24	92	87	52	63	19	18	79	89	93	90
21			72	14	4.2	4.3	23	0.44	0.67	0.39	<0.01	2.2	3.2	25	91	87	50	62	19	19	91	101	105	98
22	12.5	37.5	0	4.9	1.4	2.3	9.2	<0.01	0.02	<0.01	<0.01	4.2	28	40	68	25	20	57	16	27	92	91	101	99
23			1	7.6	1.4	4.0	13	<0.01	0.05	<0.01	0.01	1.5	21	36	89	43	29	59	10	31	92	89	99	98
24			4	8.7	1.1	6.2	16	0.01	0.10	<0.01	0.04	1.1	18	35	92	52	33	54	7	39	96	93	105	99
25			8	9.6	1.1	7.3	17	0.01	0.14	<0.01	0.08	1.0	15	32	92	60	37	54	6	41	94	100	105	98
26			24	9.9	0.58	9.7	19	0.03	0.31	<0.01	0.18	0.82	9.9	29	94	74	42	49	3	48	92	95	106	97
27			48	9.9	0.56	11	22	0.08	0.64	0.08	<0.01	1.1	8.5	26	92	77	48	46	3	51	90	94	109	96
28			72	10	0.66	11	23	0.11	0.73	0.08	<0.01	1.0	6.4	26	92	83	48	46	3	51	94	100	106	99
29	–	50.0	0	–	–	4.2	5.5	–	0.02	–	0.01	–	42	44	–	17	11	–	–	100	95	–	100	100
30			1	–	–	6.9	8.4	–	0.04	–	0.02	–	37	41	–	26	18	–	–	100	93	–	102	99
31			4	–	–	10	11	–	0.08	–	0.05	–	30	38	–	40	23	–	–	100	96	–	102	99
32			8	–	–	13	15	–	0.15	–	0.13	–	26	37	–	48	26	–	–	100	>99	–	104	103
33			24	–	–	17	17	–	0.29	–	0.22	–	17	32	–	66	37	–	–	100	94	–	103	99
34			48	–	–	19	18	–	0.60	–	<0.01	–	14	31	–	73	39	–	–	100	94	–	103	99
35			72	–	–	18	22	–	0.82	–	0.01	–	15	28	–	71	44	–	–	100	>99	–	103	102

[a]Reaction conditions: either or both of MeOH and 1-BuOH 50 mmol in total; CeO_2 2.0 mmol; 2-CP 50 mmol; acetonitrile 100 mmol; CO_2 5.0 MPa (r.t.); 393 K; 0–72 h.

[b]Abbreviations in this table: MeOH = methanol; 1-BuOH = 1-butanol; BMC = butyl methyl carbonate; DMC = dimethyl carbonate; DBC = dibutyl carbonate; PA = picolinamide; MP = methyl picolinate; BP = butyl picolinate; MC = methyl carbamate; BC = butyl carbamate; 2-CP = 2-cyanopyridine.

Table S16. Detailed data for Figure S10 (time courses for synthesis of butyl methyl carbonate (BMC) from MeOH, 1-BuOH, and CO_2 over CeO_2 catalyst at the MeOH/1-BuOH molar ratio of 25:25 with the different amount of 2-CP).[a,b]

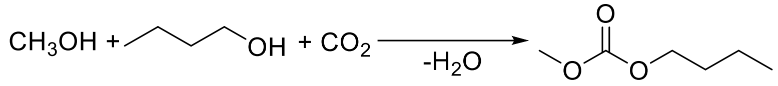

Butyl methyl carbonate (BMC)

Entry	2-CP	*t*	Product amount [mmol]								Recovered reactant [mmol]			Conversion [%]			Distribution [%]			PA sel.	Balance [%]		
	[mmol]	[h]	BMC	DMC	DBC	PA	MP	BP	MC	BC	MeOH	1-BuOH	2-CP	MeOH	1-BuOH	2-CP	BMC	DMC	DBC	[%]	MeOH	1-BuOH	2-CP
1	25	0	5.4	3.4	1.1	11	0.02	0.02	0.02	<0.01	10	17	14	58	30	44	55	35	11	97	91	100	99
2		1	8.5	4.8	1.8	16	0.03	0.06	0.05	<0.01	4.8	13	9.4	81	46	63	56	32	12	99	90	102	100
3		4	10	4.9	2.3	18	0.06	0.15	0.09	<0.01	3.5	10	6.8	86	59	73	59	28	13	99	93	101	100
4		8	12	4.3	2.8	19	0.10	0.26	0.15	<0.01	3.2	8.4	5.8	87	67	77	62	23	15	96	94	102	98
5		24	12	3.5	3.4	19	0.24	0.49	0.28	<0.01	3.1	6.3	5.0	88	75	80	64	18	18	96	91	103	100
6		48	12	3.0	3.7	19	0.34	0.71	0.37	<0.01	2.9	5.5	5.3	88	78	79	64	16	20	95	89	103	100
7		72	12	3.5	3.6	20	0.49	0.81	0.50	<0.01	3.3	5.0	4.2	87	80	83	63	18	19	95	92	102	101
8	50	0	6.2	4.6	1.2	12	0.02	0.02	<0.01	<0.01	9.1	17	37	64	32	26	52	38	10	94	96	102	98
9		1	9.4	5.6	2.0	17	0.03	0.05	<0.01	0.01	3.9	13	32	85	49	35	55	33	12	95	97	104	98
10		4	11	4.9	2.6	18	0.04	0.09	<0.01	0.03	2.5	10	31	90	59	38	58	27	14	94	92	105	98
11		8	12	5.2	3.0	20	0.05	0.14	0.05	0.06	2.4	8.1	29	90	68	41	59	26	15	95	100	105	98
12		24	14	4.0	4.1	21	0.15	0.33	0.04	0.13	1.9	4.4	28	93	83	45	63	18	19	92	96	107	97
13		48	12	3.8	3.5	21	0.25	0.23	0.24	0.25	1.9	3.3	24	92	87	52	63	19	18	79	89	93	90
14		72	14	4.2	4.3	23	0.44	0.67	0.39	<0.01	2.2	3.2	25	91	87	50	62	19	19	91	101	105	98

[a]Reaction conditions: MeOH 25 mmol; 1-BuOH 25 mmol; CeO_2 2.0 mmol; 2-CP 25 or 50 mmol; acetonitrile 100 mmol; CO_2 5.0 MPa (r.t.); 393 K; 0–72 h.

[b]Abbreviations in this table: MeOH = methanol; 1-BuOH = 1-butanol; BMC = butyl methyl carbonate; DMC = dimethyl carbonate; DBC = dibutyl carbonate; PA = picolinamide; MP = methyl picolinate; BP = butyl picolinate; MC = methyl carbamate; BC = butyl carbamate; 2-CP = 2-cyanopyridine.

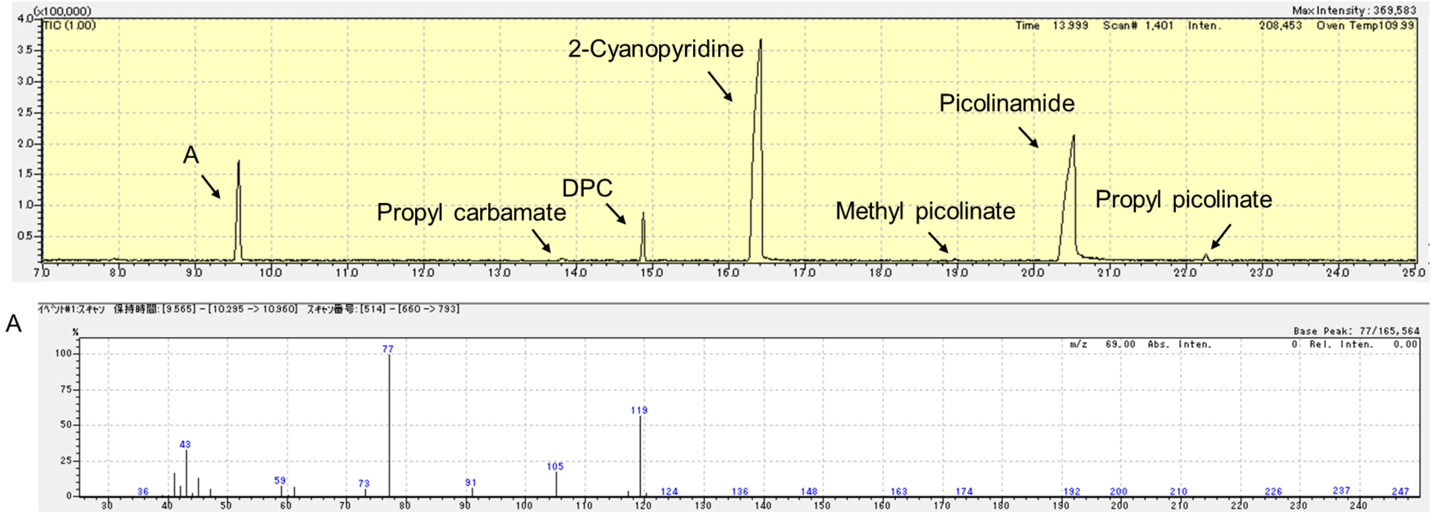

Figure S1. Total ion chromatogram and mass spectrum of peak A for the reaction mixture of the direct synthesis of MPC, recorded by GC-MS(CI).
Reaction conditions: methanol 25 mmol; 1-propanol 25 mmol; CeO_2 2.0 mmol; 2-CP 50 mmol; acetonitrile 100 mmol; CO_2 5 MPa (r.t.); 393 K; 24 h.
Column for GC-MS: CP-Sil 5 CB capillary column (ø0.25 mm × 50 m).

Assignments for mass spectrum in Figure S1

Methyl propyl carbonate (MPC, M = 118 g mol^{-1}): 119 ($[M + H]^+$), 105 ($[M + H – CH_2]^+$), 91 ($[M + H – CH_2CH_2]^+$), 77 ($[M + H – CH_2CHCH_3]^+$), 59 ($[M + H – CH_3CH_2CH_2OH]^+$).

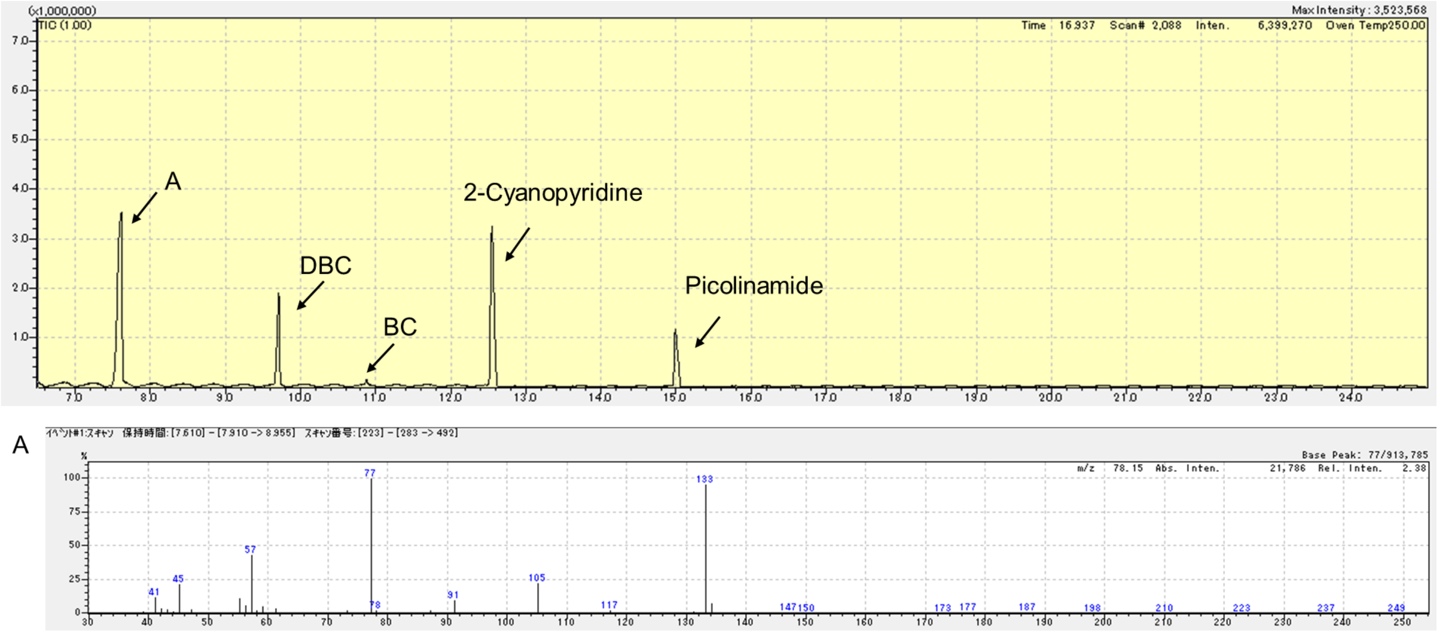

Figure S2. Total ion chromatogram and mass spectrum of peak A for the reaction mixture of the direct synthesis of BMC, recorded by GC-MS(CI).

Reaction conditions: methanol 25 mmol; 1-butanol 25 mmol; CeO_2 2.0 mmol; 2-CP 50 mmol; acetonitrile 100 mmol; CO_2 5 MPa (r.t.); 393 K; 24 h.

Column for GC-MS: TC-WAX capillary column (ø0.25 mm × 30 m).

Assignments for mass spectrum in Figure S2

Butyl methyl carbonate (BMC, M = 132 g mol^{-1}): 133 ($[M + H]^+$), 117 ($[M + H – CH_4]^+$), 105 ($[M + H – CH_2CH_2]^+$), 91 ($[M + H – CH_2CHCH_3]^+$), 77 ($[M + H – CH_2CHCH_2CH_3]^+$), 57 ($[M + H – CH_3OCOOH]^+$).

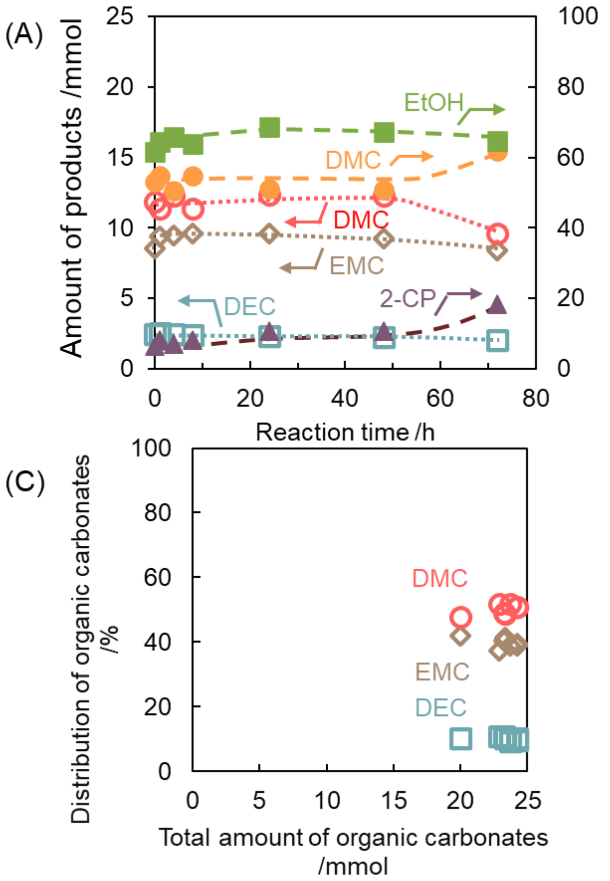

Figure S3. Time courses for transesterification between DMC and EtOH to synthesize ethyl methyl carbonate (EMC) over CeO_2 (A and C) in the presence of 2-CP and (B and D) in the absence of 2-CP. The panels A and B represent the time courses of amount of products. The panels C and D exhibit the distribution of the three organic carbonates as a function of total amount of organic carbonates (C) with 2-CP and (D) without 2-CP.

Reaction conditions: DMC 25 mmol; EtOH 25 mmol; CeO_2 2.0 mmol; 2-CP 0 or 50 mmol; acetonitrile 100 mmol; Ar 5.0 MPa (r.t.); 393 K; 0–72 h.

Detailed data are summarized in Table S8.

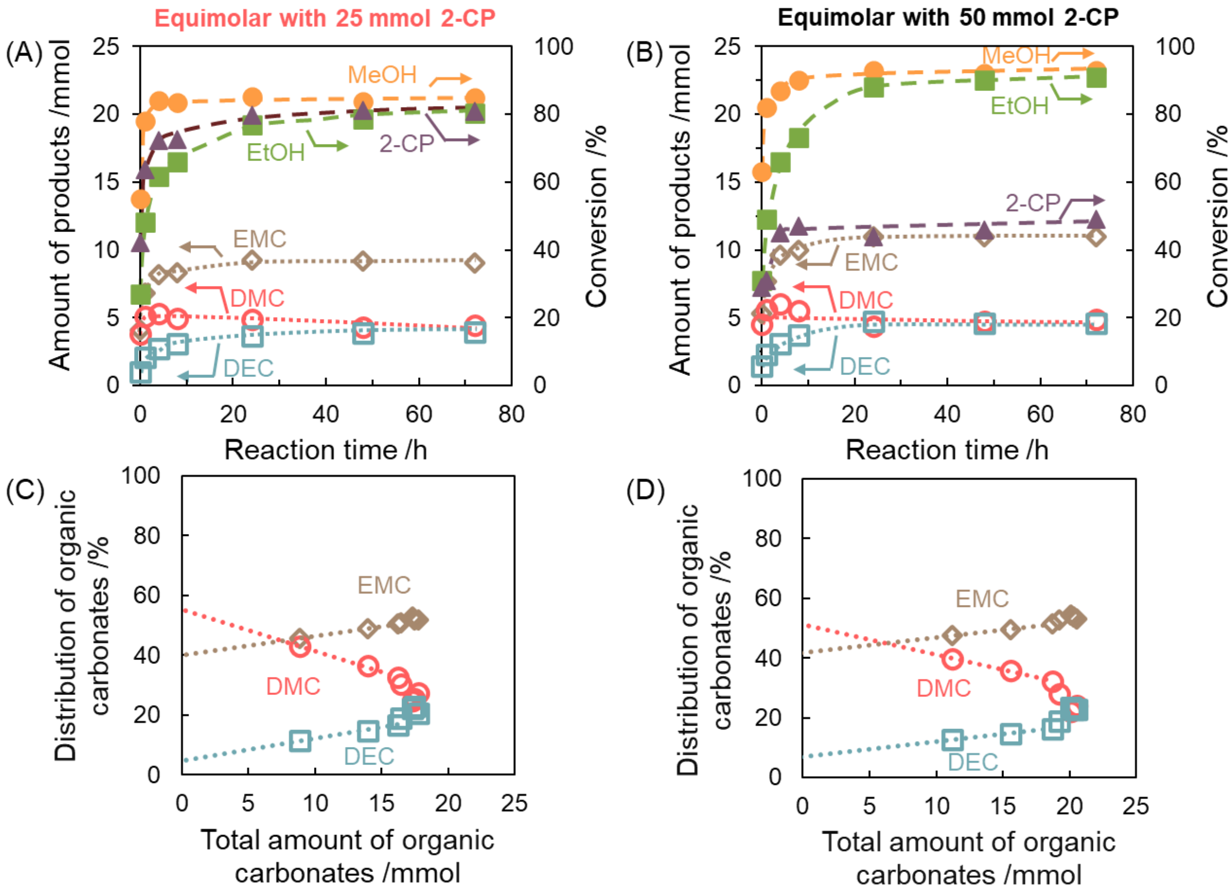

Figure S4. Time courses for synthesis of ethyl methyl carbonate (EMC) from MeOH, EtOH, and CO_2 over CeO_2 catalyst at the MeOH/EtOH molar ratio of 25:25 with the different amount of 2-CP: (A) 25 mmol and (B) 50 mmol. Parametric plot for the distribution of three organic carbonates as a function of their total amount at the different amount of 2-CP: (C) 25 mmol and (D) 50 mmol.

Reaction conditions: MeOH 25 mmol; EtOH 25 mmol; CeO_2 2.0 mmol; 2-CP 25 or 50 mmol; acetonitrile 100 mmol; CO_2 5.0 MPa (r.t.); 393 K; 0–72 h.

Detailed data are summarized in Table S9.

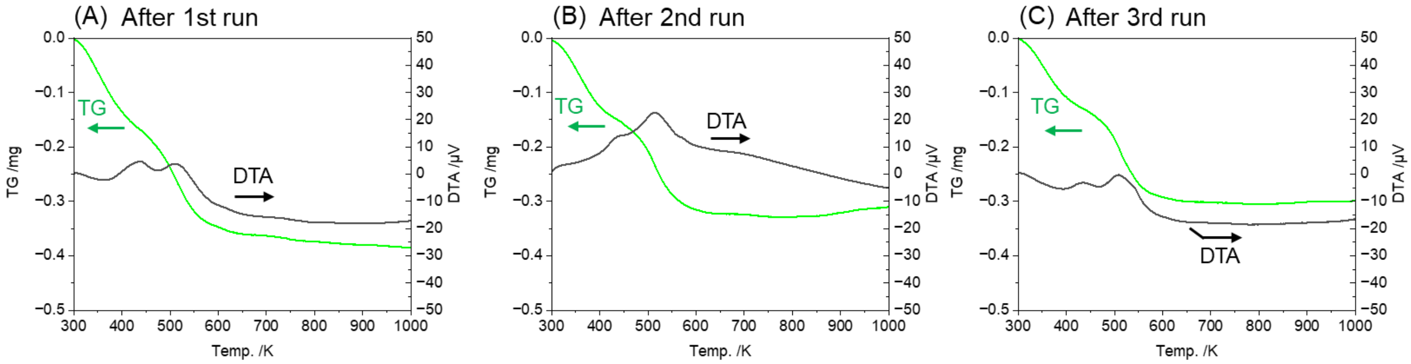

Figure S5. TG-DTA profiles for the spent CeO_2 catalyst after each run in the reuse test (Table S10).
Measurement conditions: *ca.* 10 mg of spenct catalyst; air flow 30 mL min^{-1}; ramp rate 10 K min^{-1}.
Reaction conditions: MeOH 25 mmol; EtOH 25 mmol; CeO_2 2.0 mmol; 2-CP 50 mmol; acetonitrile 100 mmol; CO_2 5.0 MPa (r.t.); 393 K; 0 h.
The spent catalyst was separated from the reaction mixture by centrifugation and decantation, dried at 333 K overnight, and calcined in air at 873 K for 3 h.

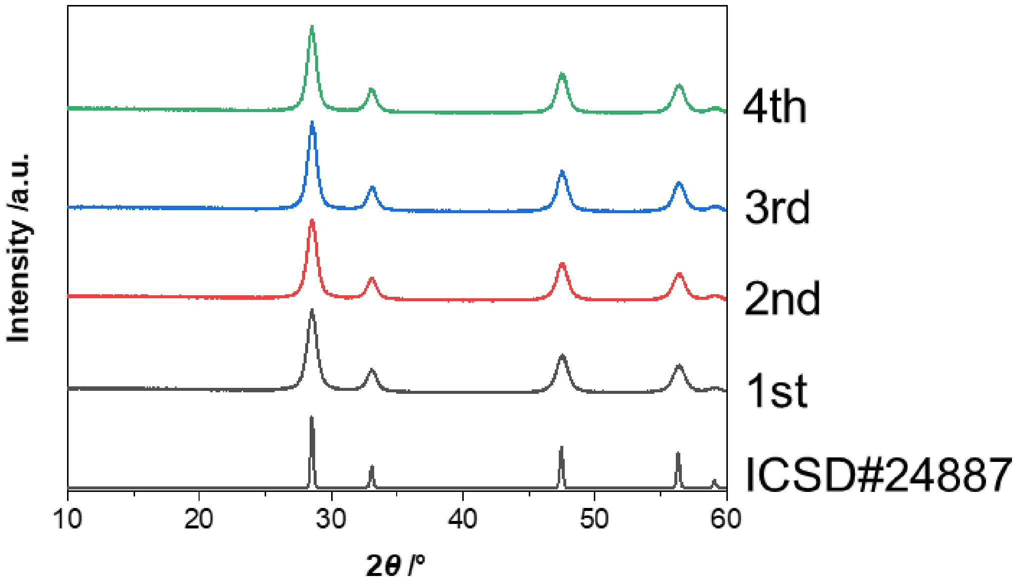

Figure S6. XRD patterns of the spent CeO_2 catalyst after each run and subsequent calcination in air at 873 K in the reuse test (Table S10).
Reaction conditions: MeOH 25 mmol; EtOH 25 mmol; CeO_2 2.0 mmol; 2-CP 50 mmol; acetonitrile 100 mmol; CO_2 5.0 MPa (r.t.); 393 K; 0 h.
The spent catalyst was separated from the reaction mixture by centrifugation and decantation, dried at 333 K overnight, and calcined in air at 873 K for 3 h.

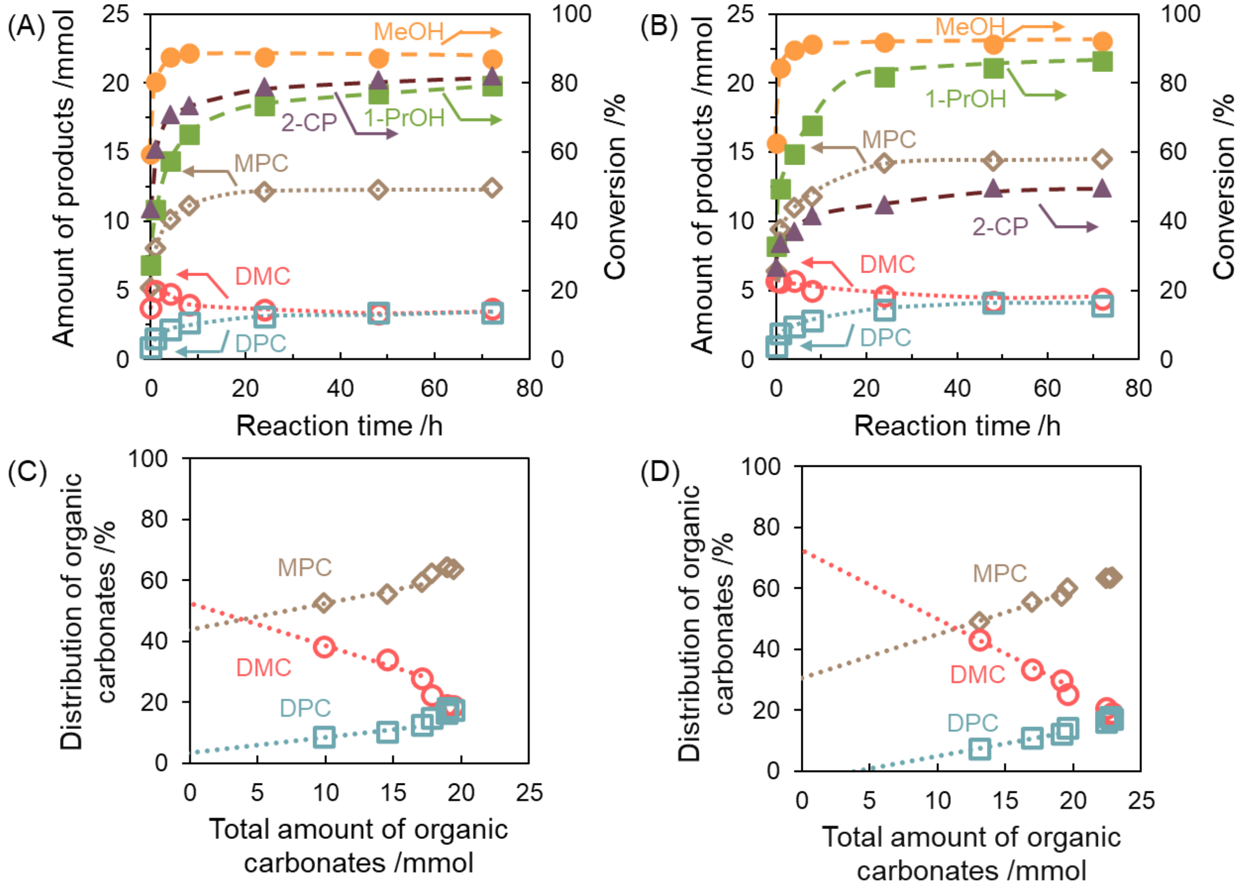

Figure S7. Time courses for synthesis of methyl propyl carbonate (MPC) from MeOH, 1-PrOH, and CO_2 over CeO_2 catalyst at the MeOH/1-PrOH molar ratio of 25:25 with the different amount of 2-CP: (A) 25 mmol and (B) 50 mmol. Parametric plot for the distribution of three organic carbonates as a function of their total amount at the different amount of 2-CP: (C) 25 mmol and (D) 50 mmol.

Reaction conditions: MeOH 25 mmol; 1-PrOH 25 mmol; CeO_2 2.0 mmol; 2-CP 25 or 50 mmol; acetonitrile 100 mmol; CO_2 5.0 MPa (r.t.); 393 K; 0–72 h.

Detailed data are summarized in Table S12.

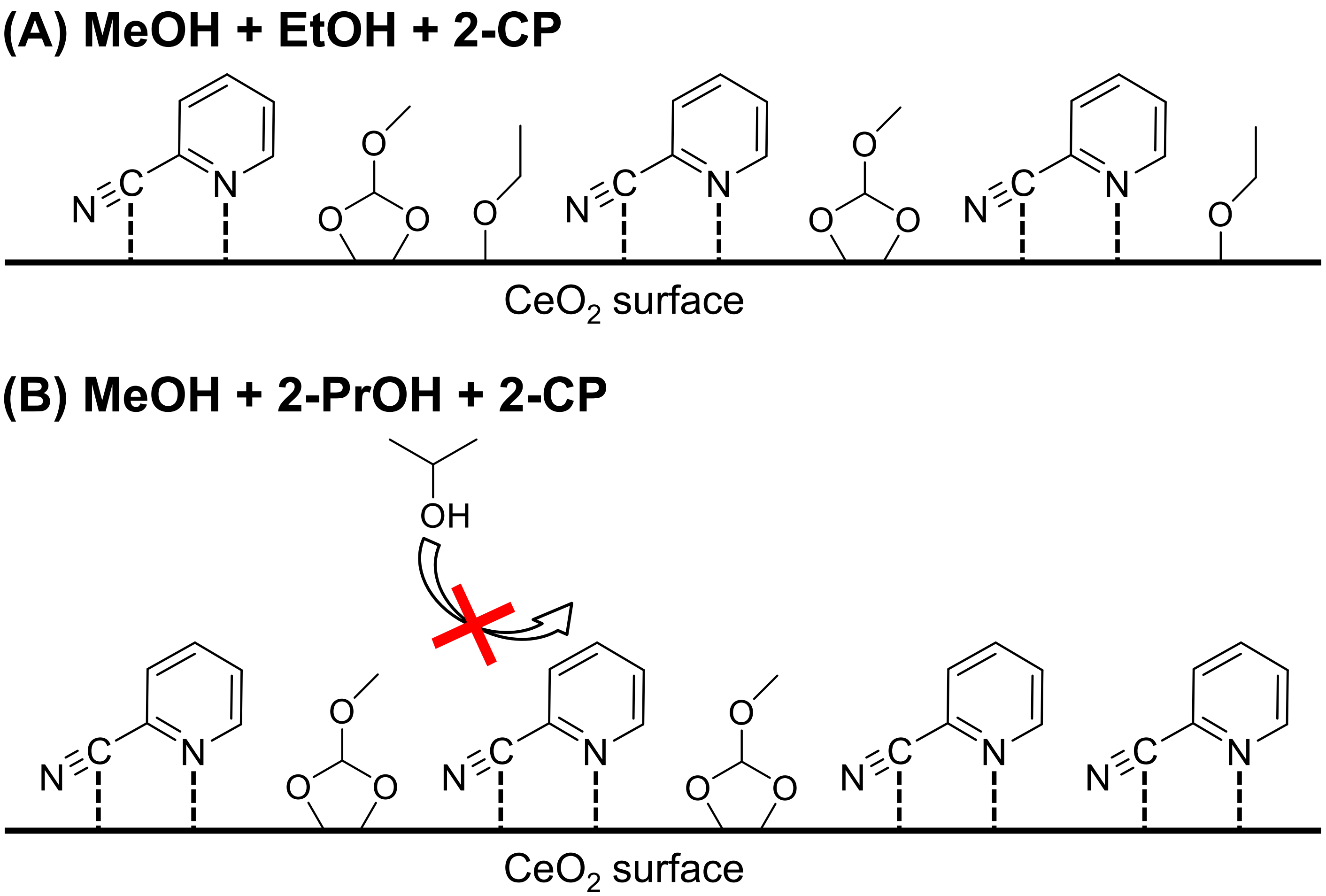

Figure S8. Inhibitory effect of 2-CP adspecies on the synthesis of organic carbonates from alcohols and CO_2 over CeO_2 catalyst, suggested from our previous study.[S33]

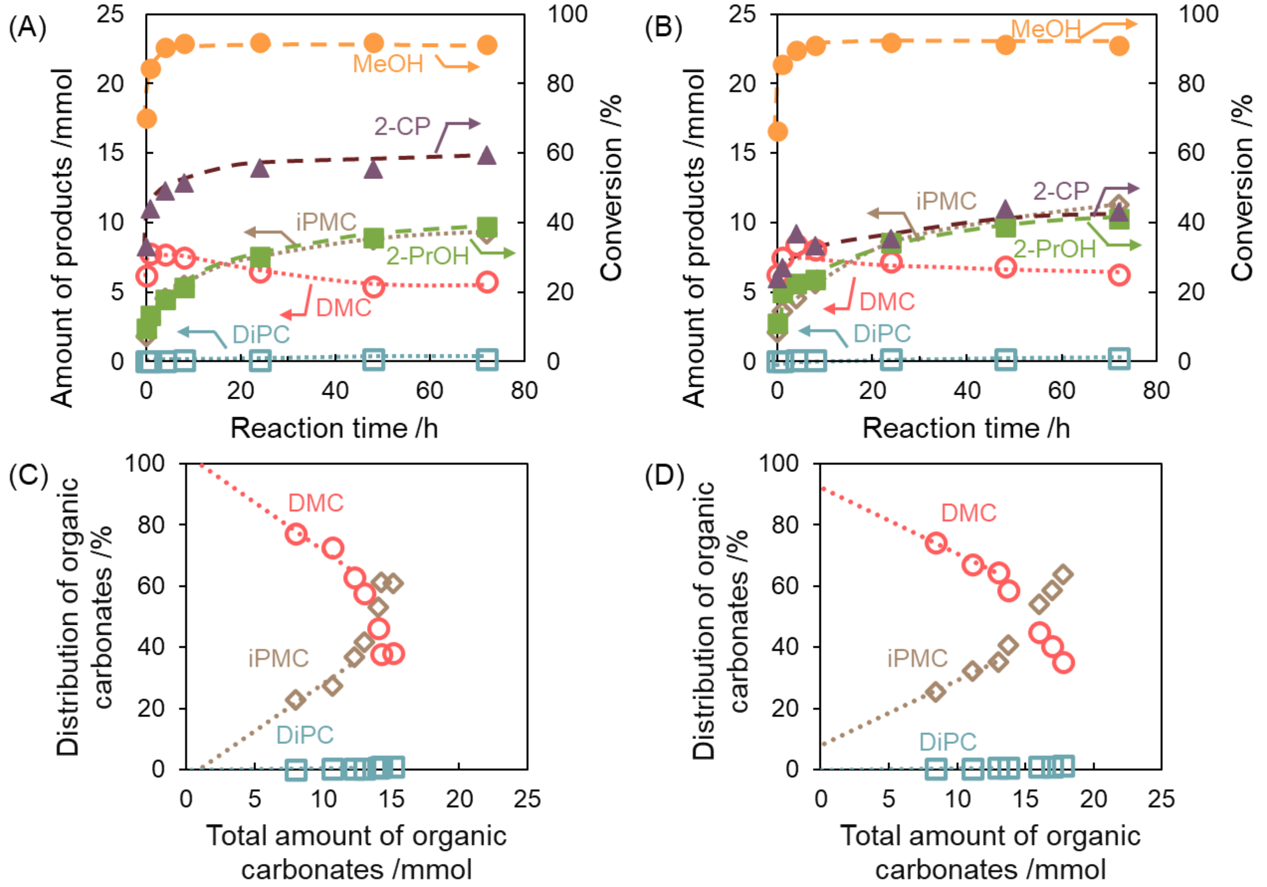

Figure S9. Time courses for synthesis of isopropyl methyl carbonate (iPMC) from MeOH, 2-PrOH, and CO_2 over CeO_2 catalyst at the MeOH/2-PrOH molar ratio of 25:25 with the different amount of 2-CP: (A) 25 mmol and (B) 50 mmol. Parametric plot for the distribution of three organic carbonates as a function of their total amount at the different amount of 2-CP: (C) 25 mmol and (D) 50 mmol.

Reaction conditions: MeOH 25 mmol; 2-PrOH 25 mmol; CeO_2 2.0 mmol; 2-CP 25 or 50 mmol; acetonitrile 100 mmol; CO_2 5.0 MPa (r.t.); 393 K; 0–72 h.

Detailed data are summarized in Table S14.

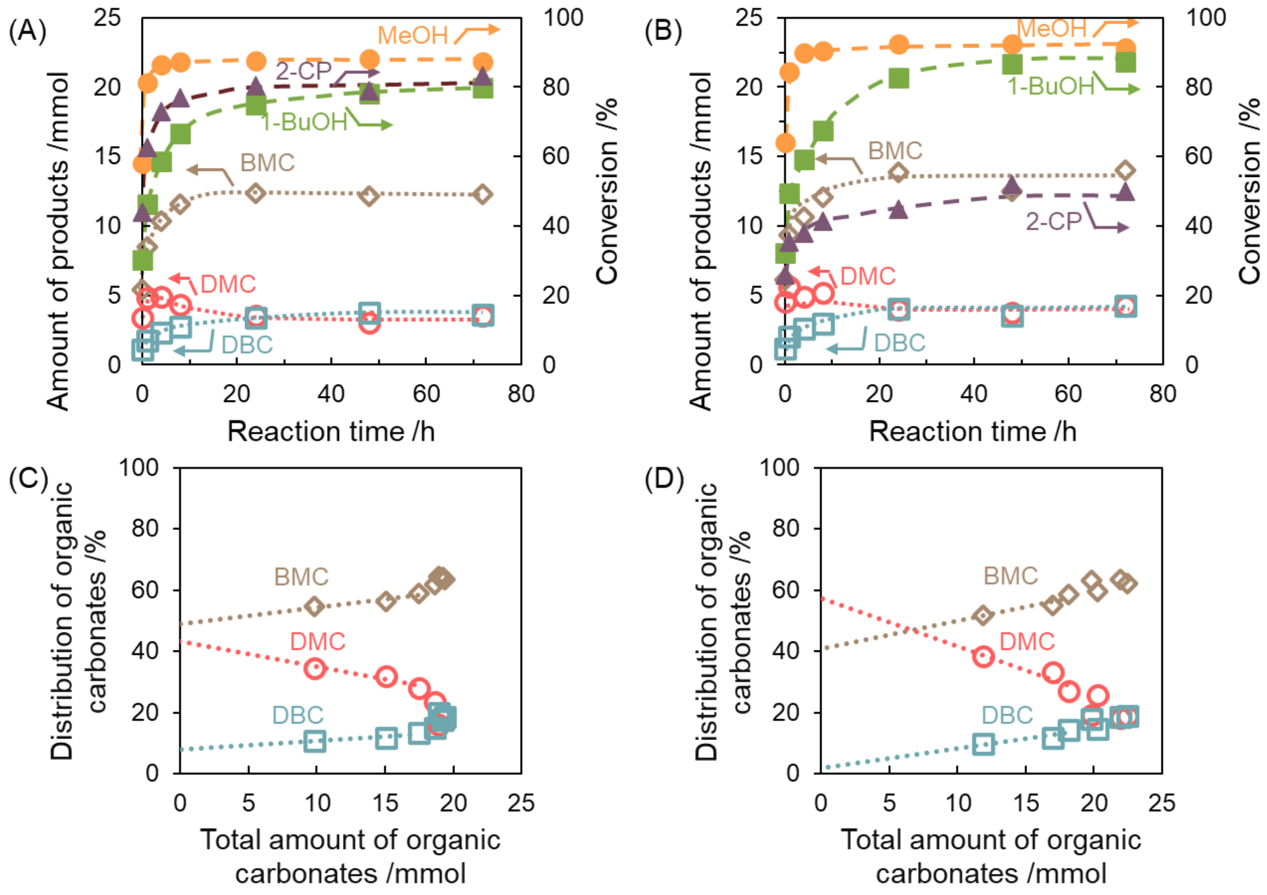

Figure S10. Time courses for synthesis of butyl methyl carbonate (BMC) from MeOH, 1-BuOH, and CO_2 over CeO_2 catalyst at the MeOH/1-BuOH molar ratio of 25:25 with the different amount of 2-CP: (A) 25 mmol and (B) 50 mmol. Parametric plot for the distribution of three organic carbonates as a function of their total amount at the different amount of 2-CP: (C) 25 mmol and (D) 50 mmol.

Reaction conditions: MeOH 25 mmol; 1-BuOH 25 mmol; CeO_2 2.0 mmol; 2-CP 25 or 50 mmol; acetonitrile 100 mmol; CO_2 5.0 MPa (r.t.); 393 K; 0–72 h.

Detailed data are summarized in Table S16.

Supplementary references

[S1] J. Lv, H. Cai, Y. Guo, W. Liu, N. Tao, H. Wang, J. Liu, *ChemistrySelect* **2019**, *4*, 7366–7370.

[S2] J. Chen, J. Xie, X. Chen, R. Dong, X.-H. Ge, T. Qiu, *Green Chem.* **2024**, *26*, 10500–10511.

[S3] X. Chen, Y. Cai, R. Dong, H. Huang, Z. Huang, T. Qiu, J. Chen, *Chem. Eng. Sci.* **2024**, *300*, 120572.

[S4] B. B. Kulkarni, S. K. R. Velagala, S. P. Maradur, *Ind. Eng. Chem. Res.* **2024**, *63*, 9761–9771.

[S5] K. S. Kanakikodi, S. R. Churipard, A. B. Halgeri, S. P. Maradur, *Sci. Rep.* **2020**, *10*, 13103.

[S6] Y. Zhou, Q. Jin, Z. Gao, H. Guo, H. Zhang, X. Zhou, *RSC Adv.* **2014**, *4*, 7013–7018.

[S7] H. Zhao, H. Chen, M. Y. Zhang, Y. Yang, Z. Yang, P. Ma, J. Niu, J. Wang, *Dalton Trans.* **2024**, *53*, 5562–5566.

[S8] L. Desidery, S. Chaemcheun, M. Yusubov, F. Verpoort, *Catal. Commun.* **2018**, *104*, 82–85.

[S9] I. Zielinska-Nadolska, K. Warmuzinski, J. Richter, *Catal. Today* **2006**, *114*, 226–230.

[S10] Z. Zhao, M. Liu, Y. Wang, Z. Yan, G. Xu, J. Guo, L. Shi, *React. Chem. Eng.* **2023**, *8*, 1654–1664.

[S11] P. Wang, S. Liu, X. Cui, F. Shi, *Catal. Sci. Technol.* **2024**, *14*, 6947–6955.

[S12] Z. Qi, S. Li, Y. Cai, R. Cui, J. Chen, C. Ye, T. Qiu, *Fuel* **2023**, *334*, 126659.

[S13] H. Wang, W. Liu, Y. Wang, N. Tao, H. Cai, J. Liu, J. Lv, *Ind. Eng. Chem. Res.* **2020**, *59*, 5591–5600.

[S14] R. Srivastava, D. Srinivas, P. Ratnasamy, *J. Catal.* **2006**, *241*, 34–44.

[S15] Q. Gu, J. Fang, Z. Xu, W. Ni, K. Kong, Z. Hou, *New J. Chem.* **2018**, *42*, 13054–13064.

[S16] D. Yanmin, C. Xingquan, Z. Chunxiang, Z. Tiansheng, *J. Mol. Catal. A: Chem.* **2010**, *331*, 125–129.

[S17] G. Trapasso, C. Salaris, M. Reich, E. Logunova, C. Salata, K. Kümmerer, A. Figoli, F. Aricò, *Sustainable Chem. Pharm.* **2022**, *26*, 100639.

[S18] S.-H. Pyo, R. Hatti-Kaul, *Adv. Synth. Catal.* **2016**, *358*, 834–839.

[S19] S. Kumar, S. L. Jain, *New J. Chem.* **2013**, *37*, 3057–3061.

[S20] S. Jin, A. J. Hunt, J. H. Clark, C. R. McElroy, *Green Chem.* **2016**, *18*, 5839–5844.

[S21] T. Tabanelli, S. Cailotto, J. Strachan, A. F. Masters, T. Maschmeyer, A. Perosa, F. Cavani, *Catal. Sci. Technol.* **2018**, *8*, 1971–1980.

[S22] B. Zhang, G. Ding, H. Zheng, Y. Zhu, *Appl. Catal. B: Environ.* **2014**, *152–153*, 226–232.

[S23] D. Chevella, A. K. Macharla, R. Banothu, K. S. Gajula, V. Amrutham, M. Boosa, N. Nama, *Green Chem.* **2019**, *21*, 2938–2945.

[S24] K. S. Kanakikodi, N. Kulal, K. S. Subramanya, M. S. Puneethkumar, B. B. Kulkarni, G. V. Shanbhag, S. P. Maradur, *Mol. Catal.* **2024**, *552*, 113667.

[S25] K. Liu, C. Liu, *ChemistrySelect* **2021**, *6*, 10548–10553.

[S26] X. Hu, J. Wang, M. Mei, Z. Song, H. Cheng, L. Chen, Z. Qi, *Chem. Eng. J.* **2021**, *413*, 127469.

[S27] E. S. Domalski, *J. Phys. Chem. Ref. Data* **1972**, *1*, 221–277.

[S28] National Institute of Standards and Technology, U.S. Department of Commerce. NIST Chemistry WebBook, Standard Reference Database Number 69. https://webbook.nist.gov/chemistry/ (accessed 25 December 2025).

[S29] W. V. Steele, R. D. Chirico, S. E. Knipmeyer, A. Nguyen, N. K. Smith, *J. Chem. Eng. Data* **1997**, *42*, 1037–1052.

[S30] M. Månsson, *J. Chem. Thermodyn.* **1972**, *4*, 865–871.

[S31] P. Franke, *J. Electrochem. Soc.* **2025**, *172*, 070520.

[S32] S. Huang, B. Yan, S. Wang, X. Ma, *Chem. Soc. Rev.* **2015**, *44*, 3079–3116.

[S33] W. Sun, P. Li, M. Yabushita, Y. Nakagawa, Y. Wang, A. Nakayama, K. Tomishige, *ChemSusChem* **2023**, *16*, e202300768.
